# Triclabendazole suppresses cellular levels of glycosaminoglycan—A potential therapeutic agent for mucopolysaccharidoses and related diseases

**DOI:** 10.1016/j.isci.2025.113118

**Published:** 2025-07-18

**Authors:** Seigo Terawaki, Filipp Vasilev, Viktoriia Sofronova, Misa Tanaka, Yoshiko Mori, Rina Iwata, Takahito Moriwaki, Toshiharu Fujita, Nadezhda Maksimova, Takanobu Otomo

**Affiliations:** 1 Department of Molecular and Genetic Medicine, Kawasaki Medical School, Kurashiki 701-0192, Japan; 2 Laboratory of Molecular Medicine and Human Genetics, North-Eastern Federal University, Yakutsk, Sakha Republic 677013, Russia; 3 Department of Clinical Genomics, Saitama Medical University, Saitama 350-0495, Japan

**Keywords:** Health sciences, Medicine, Medical specialty, Clinical genetics, Natural sciences, Biological sciences, Physiology, Pathophysiology

## Abstract

Mucopolysaccharidosis-plus syndrome (MPSPS) is an autosomal recessive inherited disorder of mucopolysaccharide metabolism with a severe clinical course. The causative gene *VPS33A* regulates membrane trafficking, including autophagy and endocytosis. However, how the patient-specific mutation impacts VPS33A function is unknown. We have contrived an experimental method utilizing flow cytometry to evaluate protein levels required for certain cellular functions, named DEFAC, and concluded that the mutant VPS33A has a comparable function in autophagy to the wild-type at the molecular level. There is no specific treatment for MPSPS that is not due to lysosomal enzyme deficiencies or known VPS33A functions. We screened the FDA-approved drug library and identified triclabendazole as a potential curative for MPSPS. Triclabendazole reduced mucopolysaccharide in MPSPS and Mucopolysaccharidosis model cells and represented therapeutic effects on MPSPS model mice. These results suggest that triclabendazole is a widely applicable therapeutic not only to MPSPS but also to related diseases with mucopolysaccharide accumulation.

## Highlights

• Triclabendazole is a candidate drug for mucopolysaccharide accumulating diseases
• DEFAC method can evaluate protein expression and cellular function in single cells
• MPS plus syndrome specific mutant VPS33A p.R498W retains function in autophagy

## Graphical abstract

**Figure:**
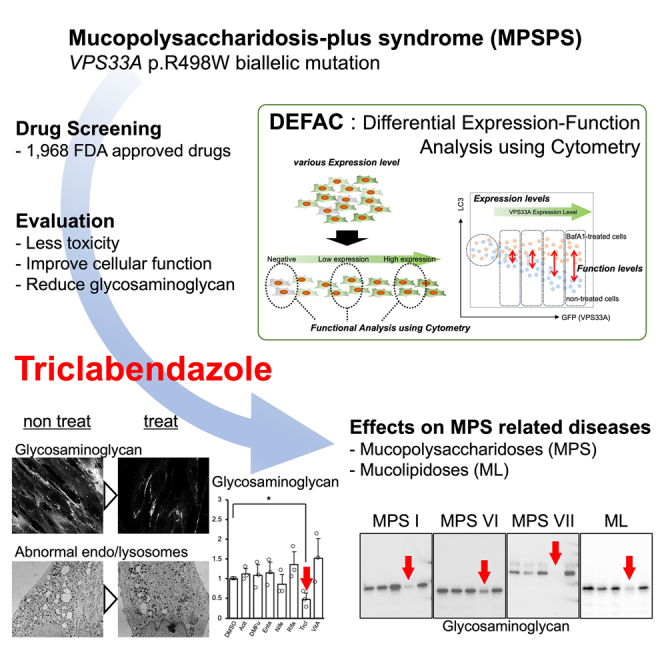

## Introduction

Mucopolysaccharidosis-plus syndrome (MPSPS, OMIM #617303)^1^^,^^2^ is defined as a disease with additional symptoms to mucopolysaccharidoses (MPS); in addition to the symptoms observed in MPS and mucolipidoses,^3^^,^^4^ such as accumulation of mucopolysaccharides (also known as glycosaminoglycans, GAGs), systemic bone deformities named dysostosis multiplex, and characteristic facial features, MPSPS presents “plus” symptoms of renal failure and hematopoietic failure, with a severe life expectancy of 20 months on average.^5^^,^^6^^,^^7^ Patients are predominantly found in the Republic of Sakha in the Russian Far East region, but also two patients of siblings in Turkey,^2^ and one patient in Italy^8^ were reported. Currently, there is no treatment other than symptomatic therapy for MPSPS.

MPSPS is caused by the specific variant p.R498W (NM_022916.6(VPS33A):c.1492C>T (p.Arg498Trp)) of the gene *VPS33A* in an autosomal recessive manner.^1^ MPSPS shows an accumulation of glycosaminoglycans in blood and urine that is clinically similar to lysosomal storage diseases such as MPS and mucolipidoses, however, there is no deficiency in the activity of lysosomal enzymes in MPSPS.^1^ Lysosomes contain tens of acidic hydrolytic enzymes and establish the basis of a large part of the intracellular degradation system.^9^^,^^10^ Intracellular substrates are transported by autophagosomes, and extracellular substrates by endosomes. These substrate-containing transport vesicles eventually fuse with lysosomes, and the contents are digested by hydrolytic enzymes within the lysosome.^9^^,^^11^^,^^12^ The causative gene of MPSPS, *VPS33A*, codes a component molecule of tethering complexes such as HOPS, CORVET, and hybrid types of the two that facilitate fusion between endosomes, autophagosomes, and lysosomes.^13^^,^^14^^,^^15^^,^^16^^,^^17^^,^^18^ Considering that the majority of GAGs are taken up by endocytosis and undergo degradation in endosomes and lysosomes,^19^ mutations in VPS33A, a subunit of the tethering complexes that regulates the endocytosis process, may cause the defects of endocytosed GAGs transportation or degradation in MPSPS patient cells. Investigation using patient skin fibroblasts revealed that the autophagic process is not impaired with the p.R498W mutation,^1^ while complete depletion of the *VPS33A* gene disrupts autophagy. The detailed mechanism of how the p.R498W mutation in VPS33A is involved in the pathogenesis and pathophysiology of MPSPS is still unclear.

## Results

### p.R498W mutation reduces VPS33A protein level

We first focused on the impact of the p.R498W variant, the only mutation specifically found in MPSPS patients, on the physiology of the VPS33A molecule. VPS33A is a subunit of the tethering complexes, such as HOPS or CORVET, and is known to function in autophagy in the fusion of autophagosomes and lysosomes.^13^^,^^14^^,^^15^^,^^16^ To investigate the effect of the VPS33A mutation on cellular function in the same genetic background, we established *VPS33A* knock-out (KO), *VPS33A*^*p.R498W*^ heterozygous, and homozygous knock-in (KI) HeLa cells by the CRISPR/Cas9 method as a cellular model of MPSPS (Figure S1A). As expected, the cellular level of Heparan sulfate, which accumulates in the MPSPS patient-derived skin fibroblasts and *VPS33A*-silenced HeLa cells,^1^ was increased in the homozygous KI cells as compared to wild-type (WT) cells (Figure S1B). Autophagy is a major cellular degradation system, and intracellular substrates taken up by autophagosomes are degraded by fusion with lysosomes, which is controlled by HOPS. LC3 proteins are recruited onto autophagosomes upon activation of autophagy as the lipidated form LC3-II, and thus degraded along with the incorporated substrates. Therefore, autophagy activity can be evaluated by monitoring lysosomal degradation of LC3-II, called LC3 flux, by Western blotting. Technically, first, the difference of LC3-II between the lysosome inhibitor Bafilomycin A1 (BafA1)-treated and untreated samples is calculated, and then the percentage of the LC3-II difference relative to the LC3-II in the BafA1-treated sample is determined as the relative amount of degradation.^20^^,^^21^^,^^22^

The autophagic flux of LC3 was completely abolished in *VPS33A* KO cells, while heterozygous and homozygous *VPS33A*^*p.R498W*^ KI cells represented a comparable level of autophagic flux to WT cells. In addition to LC3, p62 (SQSTM1) and NDP52, autophagy-adapter molecules involved in selective autophagy behaved similarly to LC3 (Figure S1C).^22^ These results are consistent with the previous report describing approximately doubled cellular Heparan sulfate and normal autophagic function in skin fibroblasts derived from MPSPS patients.^1^ Importantly, the amount of endogenous VPS33A in these cells was reduced in an MPSPS mutant allele-dependent manner (Figure S1C). Decreased levels of VPS33A protein with MPSPS mutations in cells have already been reported previously,^23^ and its correlation with the mechanism of MPSPS pathogenesis has been discussed. To understand why the VPS33A mutant protein level is reduced in cells, we performed a Cycloheximide (CHX) pulse-chase assay to check the stability of the protein.^24^ CHX is a specific inhibitor of protein translation in eukaryotes. By stopping cellular translation with CHX treatment and excluding the *de novo* synthesis of VPS33A protein, natural courses of WT and the mutant VPS33A degradation were monitored for up to 48 h. The results showed that the half-life of normal VPS33A was approximately 24 h, whereas the R498W mutant protein was reduced to less than 20% within 12 h after adding CHX (Figure S1D). These results suggest that the MPSPS mutation severely impairs the stability of the VPS33A protein, resulting in an imbalance between the production and the degradation of this protein in cells. Despite the critical role of VPS33A in autophagy, autophagy was maintained in *VPS33A*^*p.R498W*^ KI cells, in which the amount of VPS33A in the cells was significantly reduced. Therefore, we next investigated how much VPS33A is required for autophagy by gene silencing. We found that autophagy activity was intact even when the amount of VPS33A was less than 10% of that of untreated cells, and a decrease in activity was observed only when the amount of VPS33A was less than 2.5% (Figure S1E). This indicates that a very small amount of VPS33A is sufficient to drive autophagy. This result is consistent with the fact that autophagic activity is maintained even in the KI cells with reduced stability due to the MPSPS mutation.

Next, to assess the impact of the p.R498W mutation on the endosome system, we took a morphological approach. WT and the *VPS33A* KI HeLa cells under nutrient-rich conditions or under starvation condition that activates autophagy were observed by transmission electron microscopy (TEM). As a result, an accumulation of numerous aberrant endolysosomes, which seemed to encapsulate undigested substrates, was observed in *VPS33A* KO cells regardless of nutrient conditions (Figure S2). This abnormality of endolysosomes was not observed in both *VPS33A*^*p.R498W*^ heterozygous and homozygous KI cells (Figure S2). The autophagic impairment and morphological abnormality seem to correspond in HeLa cells.^25^^,^^26^ On the other hand, electron micrographs from the MPSPS patients’ lymphoblastoid cell lines (LCL) represented abnormal endolysosomes encapsulating some aggregated substrates (Figure S3A), although the cells maintained autophagic flux (Figure S3B). These results suggest that the p.R498W variant may have a different functional threshold impact on membrane trafficking among different tissues or cell types. MPSPS has been found to have different symptoms from the generalized mucopolysaccharidosis symptoms, such as renal failure and anemia, and these organ-specific symptoms may be attributed to the different effects of the MPSPS mutation among cell types.

### Differential expression-function analysis using cytometry (DEFAC) revealed a comparable function of p.R498W VPS33A to wild-type in autophagy

The reduced stability and quantity of cellular VPS33A protein could be one of the contributing factors to the development of MPSPS. However, it is unclear whether the p.R498W mutant protein retains the comparable function to WT per number of molecules. Both quantitative and qualitative loss of the molecule may occur in patients. Gene expression levels vary widely across tissues and cells. In the cells we examined, the MPSPS mutation reduced the protein level of VPS33A to less than 20% that of the WT, but it seemed to be sufficient to drive autophagy (Figure S1E). Nevertheless, we still considered the possibility that the MPSPS mutation might reduce the VPS33A levels below the threshold required for autophagic activity in specific cells. To investigate this possibility, we have contrived a new method for highly qualitative assessment. The method, which we named Differential Expression-Function Analysis using Cytometry (DEFAC), is an assessment of the relationship between protein expression level and function in a single cell. It allows simultaneous and highly sensitive analysis of phenotypic differences in cellular function due to differences in the expression level of the gene of interest by rescuing target gene expression with transient transduction of the gene in KO cells. This method can be applied to any experiment in which the cellular function to be evaluated can be measured by fluorescence, and we have applied it to the evaluation of autophagy activity in this study. LC3 is an indicator marker of autophagic activity because it is activated and recruited onto autophagosomes, where it is degraded along with its internalized substrate. Since treatment of cells with BafA1 stops autophagic degradation, the difference in LC3 amount with and without BafA1 treatment reflects autophagy activity (LC3 Flux Assay).^20^^,^^21^^,^^22^ Due to the nature of this experiment, single-cell analysis is not possible in the DEFAC of the current study, but it is possible to measure autophagy activity for each cell population segmented by the VPS33A protein level (Figure S4).

**Figure 1:**
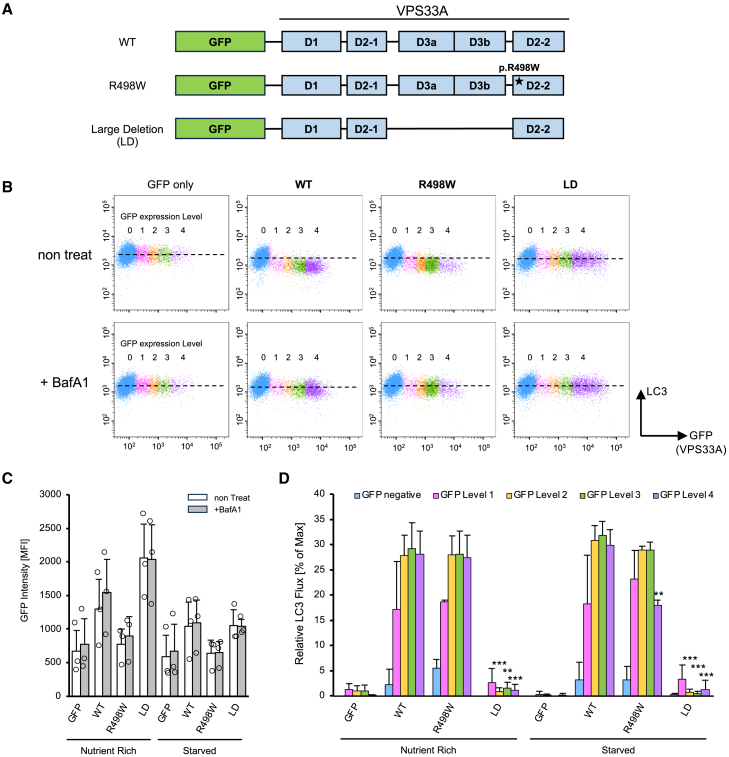
Functional equivalence of WT and R498W VPS33A, assessed by DEFAC (A) The domain structures of expression constructs for GFP-tagged *VPS33A* WT and mutants. All constructs were incorporated into the CMV promoter-driven expression vector (pcDNA3.1) and were transiently transduced to *VPS33A* KO cells. (B) Dot plot of fluorescence intensity of GFP (horizontal axis) and LC3 (vertical axis) on a two-dimensional plane. Cells were classified into five levels (Level 0–Level 4) of cell population, and each gate was differentially color-coded according to GFP fluorescence intensity. Upper row: untreated cells, Lower row: 125 nM BafA1-treated cells. The dashed line represents the mean LC3 intensity of the GFP-negative population. (C) Mean GFP intensity for the gross population of each sample. The small circles represent the data from three independent experiments, and the bar graphs represent averages and S.D. of the geometric mean fluorescence intensity (MFI) of the three experiments. (D) LC3 flux for each population from nutrient-rich and starved conditions obtained by DEFAC, both for the nutrient-rich condition and the starved condition. The fluxes of each population are shown as a relative value when all LC3 was degraded by autophagy as 100%: LC3 MFI ^BafA1+^ – LC3 MFI ^non-treat^/LC3 ^BafA1+^ MFI × 100 (%). The color of each bar matches the color of the cell population shown in the dot plot (B). Bars represent the mean and S.D. of three independent examinations. Statistical analysis was performed in a two-group comparison with a population of WT samples of the same GFP levels using Student’s *t* test. (∗∗*p* < 0.01, ∗∗∗*p* < 0.001).

We transiently expressed *GFP-labeled VPS33A* constructs (Figure 1A) in *VPS33A* KO cells and measured LC3 flux in each cell population with different expression levels of VPS33A using a flow cytometer (Figure 1B). The endogenous LC3 was fluorescently labeled with a specific antibody, and the LC3 flux per population was calculated from the average fluorescence intensity by subtracting the value of non-treated samples from BafA1-treated samples. The overall GFP fluorescent level was not affected by BafA1 treatment in each sample, which means that BafA1 does not affect the fluorescence of the expressed GFP itself (like quenching), and is a very important result for ensuring the accuracy of DEFAC (Figure 1C). As expected, LC3 flux was left completely arrested both in the *VPS33A* KO cells transfected with a mock GFP and rescued with a mutant VPS33A lacking domain 3 (Large Deletion, LD), which is required for the interaction with VPS16 in the tethering complexes.^27^^,^^28^^,^^29^ In contrast, in the cells rescued with WT VPS33A, LC3 flux recovered according to the VPS33A level (Figure 1D). From the point where GFP intensity exceeded Level 2, the effect of rescuing VPS33A for autophagy was saturated, and no further increase in exogenous *VPS33A* expression resulted in no change in LC3 flux. The LC3 flux was also recovered in the KO cells rescued with the MPSPS mutant VPS33A (Figure 1D), almost the same as the WT construct. Even in the population expressing extremely low amounts of VPS33A (GFP Level 1), LC3 flux was comparable to the population of the same level of wild-type VPS33A protein (Figures 1B and 1D). This indicates that, as far as the autophagy process is concerned, the instability of VPS33A caused by the MPSPS mutation does not affect its function and that wild-type and mutant proteins can work equally at the molecular level.

We examined the effect of the MPSPS mutations on the formation of tethering complexes by immunoprecipitation to confirm the conclusions obtained by the DEFAC experiment from another aspect. The tethering complex is composed of the products of 6 *VPS family* genes in both HOPS and CORVET (Figure S5A). Of these, VPS33A, VPS16, VPS18, and VPS11 are shared by HOPS and CORVET, but the ends of the bridge connecting vesicle and vesicle differ between HOPS (VPS41 and VPS39) and CORVET (VPS8 and VPS3; Figure S5A). Therefore, an immunoprecipitation of one of the subunits at one end of the bridge and the detection of other subunits by western blotting will tell whether the corresponding complex is formed or not.^17^ We transfected *VPS33A* KO cells with four different tagged VPS family genes that constitute the HOPS or CORVET bridge, as well as WT or the mutant *VPS33A*, and performed immunoprecipitation with VPS41 (for HOPS) and VPS8 (for CORVET) using anti-Myc tag antibody (Figures S5B and S5C). As a result, there were no problems with the HOPS formation in the mock-transfected cells, while the CORVET formation was significantly disturbed in *VPS33A* KO cells (Figure S5C). Importantly, both WT and the mutant VPS33A successfully rescued the bridge formation of CORVET. Consistent with the DEFAC result, immunoprecipitation experiments confirmed that p.R498W mutation on VPS33A did not affect the bridge formation of the tethering complexes. From these results, we concluded that the p.R498W mutation confers instability to the VPS33A protein, but the mutant can function comparably to WT VPS33A per molecule in autophagy. Therefore, it is inferred that the pathogenesis of MPSPS is due to the presence of tissues or cell types that are very sensitive to the reduced abundance of VPS33A, or a defect in VPS33A function unrelated to the tethering complex, or both.

### Triclabendazole was selected as a candidate therapeutic agent for MPSPS through screening of the FDA-approved drug library

MPSPS is a very rare disease, with only about 20 cases reported worldwide.^1^^,^^2^^,^^5^^,^^6^^,^^7^^,^^8^^,^^23^ The clinical knowledge of the pathogenesis and patient samples is limited, making the development of specific disease treatment very difficult. The previous reports,^1^^,^^23^ and current studies described above have shown not only that GAGs accumulation in MPSPS cells but the p.R498W VPS33A protein is functional as far as known, although the mutation markedly reduces the stability of VPS33A. Unlike mucopolysaccharidoses, which are caused by a deficiency of the lysosomal enzymes that degrade GAGs, enzyme replacement therapy (ERT) cannot be a treatment for MPSPS. Therefore, we decided to explore drugs that could reduce GAG accumulation without reducing the amount of mutant VPS33A protein using the MPSPS model cells.

**Figure 2:**
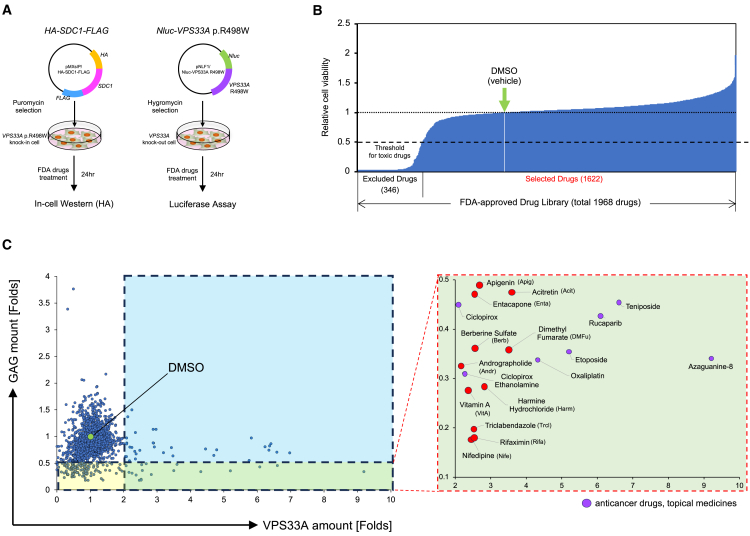
Screening of therapeutic agents for MPSPS from an FDA-approved drug library (A) Establishing the reporter cell lines for evaluating the amount of GAG (Syndecan-1, SDC1) and for the mutant VPS33A protein levels in the following drug screening. HA-tagged SDC1 expression vector and *GFP*-tagged *VPS33A*^*p.R498W*^ expression vector were stably introduced to *VPS33A*^*p.R498W*^ KI cells and *VPS33A* KO cells, respectively. (B) Toxicity checks of all drugs in the library on the reporter cell viability. The CellMask signal for the drug-treated cell was normalized to the value of DMSO as 1 (green arrow) and was plotted from the order of the lowest to the highest signal. The drugs with the signal less than 50% of the DMSO-treated samples (leftmost compartment, 346 drugs) were excluded from further analysis as highly cytotoxic drugs. (C) A dot-plot representation of the effects of the FDA-approved drugs on the GAG and VPS33A levels. The relative amount of the mutant VPS33A is plotted on the horizontal axis, while the relative GAG (SDC1) amount adjusted by cell number is plotted on the vertical axis for each drug-treated sample. The DMSO-treated sample (the coordinate 1,1) is indicated as a green dot. The lower-right quadrant, including drugs satisfying both GAG-decreasing (<0.5) and maintaining the mutant VPS33A amount (>2.0), is expanded on the right-side panel. Anticancer drugs and topical medicines shown as purple dots were excluded from the candidates in the following analysis. The other selected drugs are shown as red dots.

It has been reported that the accumulation of dermatan sulfate and heparan sulfate in the plasma or urine of MPSPS patients.^1^^,^^2^^,^^23^ So, we have generated cells that constitutively express HA- and FLAG-tagged Syndecan 1 (SDC1), a core protein of heparan sulfate,^30^ in the *VPS33A*^pR498W^ KI cells as a reporter of GAGs accumulation, since direct quantification of specific GAGs is technically difficult. In addition, we have also generated *VPS33A* KO cells with expression of *VPS33A*^*p.R498W*^ fused with *nano luciferase* (*Nluc*)^31^ to monitor its product stability (Figure 2A). A commercially available FDA-approved drug library (containing 1968 drugs) was used as the screening target. The reporter cells were seeded in 96-well plates and then were treated with the drug at a concentration of 50 μM for 24 h. The amount of SDC1 was evaluated by In-Cell Western^32^^,^^33^ with an antibody against the HA tag added to the N-terminus of SDC1, and the effect of each drug on the VPS33A mutant protein stability was determined by Luciferase activity, separately. The number of cells was estimated by staining with CellMask and used to normalize the results.

In the initial screening of the 1968 drugs, 346 drugs that decreased cell viability to less than 50% of vehicle (DMSO) were initially excluded as highly toxic agents using the CellMask signals (Figure 2B). Next, in the remaining 1622 drugs, 129 drugs suppressed GAG core protein level (SDC1) below 50% of the vehicle, 52 drugs maintained VPS33A levels above 2-fold of the vehicle, and 18 drugs that simultaneously met the 2 criteria were selected for the following selection (Figure 2C, right lower quadrant). The candidate drugs obtained from this screening could be administered to pregnant mothers and/or newborns. Therefore, we excluded 7 drugs, including anticancer drugs such as platinum-based drugs and topical medicines,^34^ although it is an arbitrary way, and then selected the remaining 11 drugs as candidates for the next step (Figure 2C, Table S1).

HeLa cells are a cell line derived from uterine cervical cancer^35^ and may be more resistant to some drugs than normal cells in the body, or may represent different responses in GAG metabolism from them. To prevent problems in clinical applications caused by the results from such artificial experiments, two lines of MPSPS patient skin fibroblasts and one line of LCL, obtained after ethical approval and consent, were used to test the safety and efficacy of the 11 candidate drugs. The effects of drugs of 24 h-treatment on skin fibroblast morphology and adherence (Figure S6A) and LCL viability (Figure S6B) were evaluated by phase-contrast microscopy and trypan blue staining, respectively. Since four drugs (Andrographolide, Apigenin, Berberine sulfate, and Harmine hydrochloride) reduced cell viability to less than 50% in both cell types, these drugs were also excluded from the candidates, and the seven drugs; acitretin, dimethyl fumarate, entacapone, nifedipine, rifaximin, triclabendazole, and vitamin A remained as candidates.

**Figure 3:**
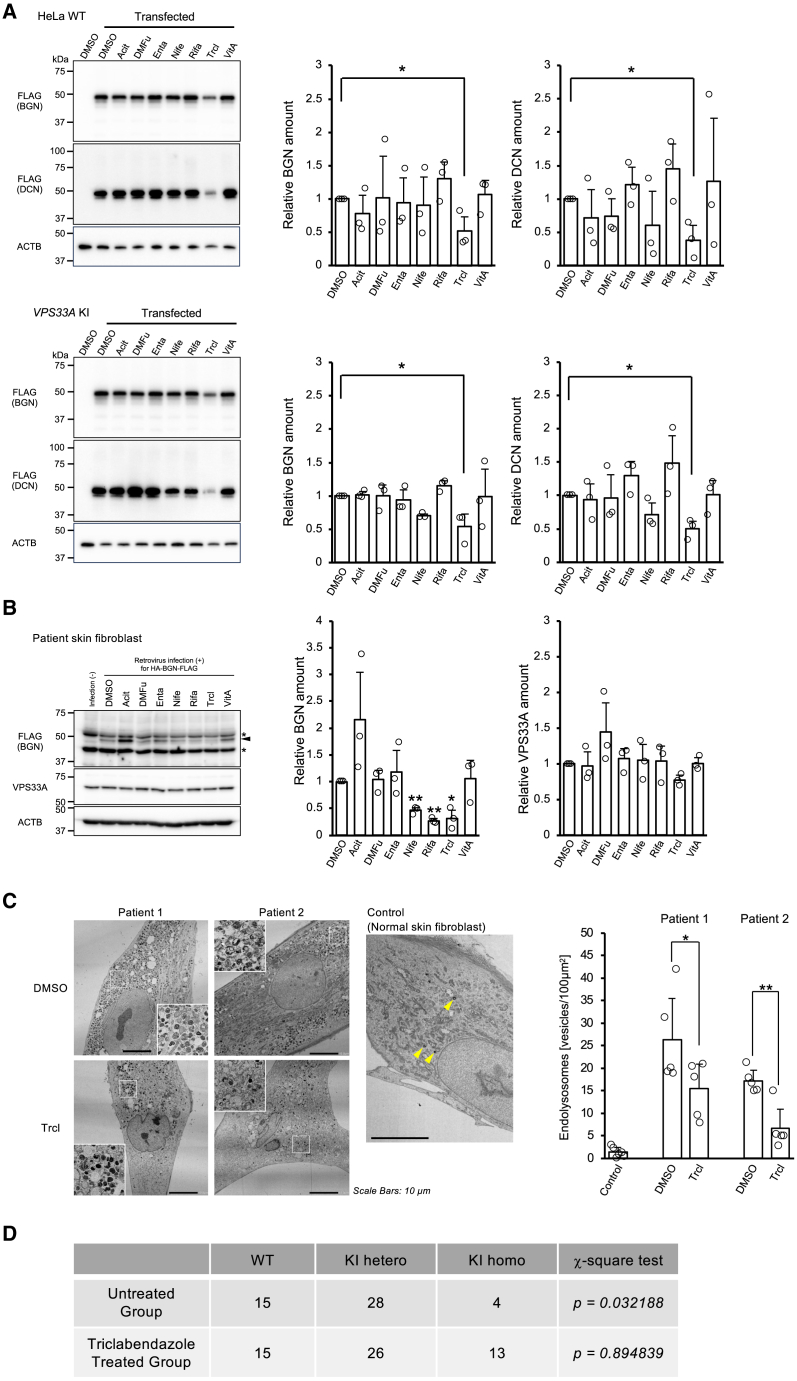
GAG-core protein reducing effects by the selected candidate drugs (A) The effect of selected drugs on the cellular levels of exogenously introduced Biglycan (BGN) or Decorin (DCN) in HeLa WT cells (upper panels) and the *VPS33A*^*p.R498W*^ KI cells (lower panels). Representative images of the western blotting are shown. Quantitative analyses of the corresponding bands from three independent experiments for BGN and the DCN are shown as average and S.D. Statistical significance was calculated only between the DMSO treatment and the triclabendazole treatment group by Welch’s *t* test. (∗*p* < 0.05). (B) A representative western blot for the reducing effects of the selected drugs on the GAG core protein levels in the patient skin fibroblasts. A arrowhead indicates the target band. An asterisk (∗) represents non-specific bands. Quantitative analyses of the western blot from three independent experiments for BGN and the VPS33A protein level are shown as average and S.D. on the right side. Statistical significance was calculated only between the DMSO treatment and the nifedipine, rifaximin, or triclabendazole -treated group by one-to-one comparison using Welch’s *t* test. (∗*p* < 0.05, ∗∗*p* < 0.01). (C) Subcellular structure changes in the patient skin fibroblasts by triclabendazole treatment. The representative transmission electron micrographs are shown. The insets show an enlarged picture of the square-marked area within the same image. A control electron micrograph from a normal skin fibroblast is displayed on the right side, which contains few endolysosomes with high electron density (yellow arrowheads). Quantitative analyses of the number of endolysosomes are shown on the right side. The bar graph represents the average and S.D. of the counts/field (small circle) from five fields. Statistical analysis was performed by comparing the DMSO and the triclabendazole -treated group using one-sided Student’s *t* test. (∗*p* < 0.05, ∗∗*p* < 0.01). (D) Therapeutic effects of triclabendazole on *Vps33a*^*p.R500W*^ KI mice embryo survival. *VPS33A* KI heterozygous male and female mice were crossed and divided into two groups at random. One group was continuously administered triclabendazole via drinking water. For each group, 6 pregnant mice were sacrificed around 18 d.p.c. and fetuses were removed for genotyping. Data for the six mice were pooled, and deviations from the expected Mendelian segregation ratio (WT: KI heterozygote: KI homozygote = 1:2:1) were analyzed with the *chi-square test.*

The GAG core protein-reducing effects of the selected seven drugs were further confirmed both in HeLa cells and the patient-derived skin fibroblasts by expressing exogenous *Biglycan* (*BGN*) and *Decorin* (*DCN*), which are core protein genes for chondroitin and dermatan sulfate, respectively,^30^ and are different reporters from the one used in the initial screening (*SDC1*). As a result, only triclabendazole showed the reducing effect of BGN and DCN (Figure 3A) in HeLa WT and the VPS33A KI cells. In contrast, 3 drugs, nifedipine, rifaximin, and triclabendazole, showed a reducing effect on BGN in patient skin fibroblasts (Figure 3B). These 3 drugs did not affect the amount of mutant VPS33A in the patient skin fibroblasts (Figure 3B). We also investigated the dose effects of these three drugs on WT and the VPS33A KI HeLa cells. The cells transfected with the tagged-*BGN* plasmid were treated with the different doses of the three drugs for 24 h. As a result, triclabendazole suppressed the amount of BGN at the lower concentrations of 10 μM and 50 μM (screening concentration), where nifedipine and rifaximin were effective only at 250 μM (Figure S7). Based on the results above, triclabendazole, which was effective at lower doses and suppressed several types of mucopolysaccharides in different cell types, was finally selected as the most promising therapeutic agent for MPSPS.

### Triclabendazole has therapeutic effects on the MPSPS patient-derived skin fibroblasts and MPSPS model mice survival

Since MPSPS patient cells show normal autophagic function, the only apparent disease phenotype is the accumulation of GAGs and abnormal intracellular structures (Figures S2 and S3A). The effect of reducing various types of GAGs core protein was confirmed during the multiple screening processes, so we decided to assess the therapeutic effect of triclabendazole on the intracellular structure abnormalities in the patient-derived skin fibroblasts. MPSPS patients' skin fibroblasts were treated with triclabendazole at 50 μM for 24 h and observed under TEM. The results showed that treatment with triclabendazole significantly reduced abnormal endolysosomes, which were highly accumulated in the MPSPS skin fibroblasts (Figure 3C). Altogether, triclabendazole reduces multiple types of GAG core proteins that may include heparan sulfate, chondroitin sulfate, dermatan sulfate, and ameliorates the abnormal accumulation of endolysosomes observed in MPSPS patient skin fibroblasts. It supports our expectation that triclabendazole is a potential therapeutic for MPSPS.

The creation of model animals is of great significance in the elucidation of pathogenesis and the development of treatments for MPSPS, where the number of cases is extremely small. We have generated *Vps33a*^*p.R500W*^ KI mice with a mutation corresponding to the human MPSPS mutation (p.R498W) using the *CRISPR/Cas9* method to understand the detailed pathogenesis and mechanism of MPSPS and to develop therapeutic strategies. Heterozygous mice were born normally, grew, and were capable of reproduction. However, mating of heterozygous pairs did not result in live homozygous mice. Homozygotes were occasionally found, although it was quite rare, as dead neonates immediately after birth, so we have concluded that the homozygous mice are embryonic lethal. Since *in vitro* cell-based experiments showed that triclabendazole was very effective in suppressing GAG core proteins, we decided to investigate its therapeutic effect *in vivo* using this mouse model.

Heterozygous male and female mice were mated so that WT, heterozygous, and homozygous fetuses were born as the same littermates. From the day of mating, 40 μg/mL of triclabendazole was added to drinking water and fed *ad libitum*. This dose was calculated based on the maximum tolerated dose of triclabendazole in humans and converted assuming that the maximum amount of water that a mouse of 20 g of body weight may consume is 5 mL per day. Even after several early experiments with triclabendazole administration, no surviving homozygote mice were born. We, therefore, sacrificed the triclabendazole -treated or untreated pregnant mice at approximately 18 d.p.c. and genotyped the viable fetuses *in utero* for statistical processing. The integrated results of the fetus genotypes from six pregnant mother mice of each group were shown (Figure 3D). In the untreated group, 15 WT, 28 heterozygote, and 4 homozygote fetuses were found. This separation ratio significantly deviated from Mendel’s laws, suggesting the premature death of the embryo at the beginning of gestation. On the other hand, in the triclabendazole -treated group, several homozygous fetuses were found, and the separation ratio of the fetus genotypes was almost matched with the theoretical Mendelian ratio (1:2:1; Figure 3D). These results indicate that triclabendazole has a therapeutic effect on extending the life span of homozygous fetuses, at least until just before birth. Although it is not yet clear whether this therapeutic effect on fetal survival is due to the suppression of GAG levels by triclabendazole, the results strongly support the possibility that this drug could be applied as a treatment for MPSPS that could act *in vivo* and also in a transplacental manner.

### Triclabendazole also acts in mucopolysaccharidosis and mucolipidosis cells

**Figure 4:**
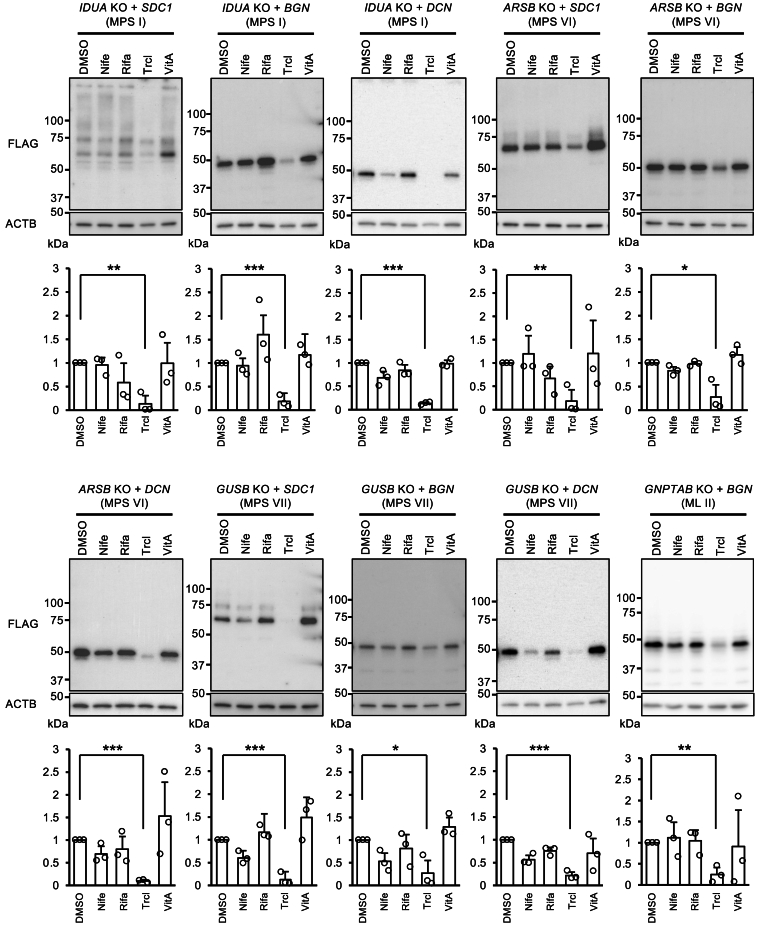
GAG core protein suppression effects of triclabendazole on multiple mucopolysaccharidosis and mucolipidosis model cells *IDUA* KO (MPS Type I), *ARSB* KO (MPS Type VI), *GUSB* KO (MPS VII), and *GNPTAB* KO (ML II/I-cell disease) model HeLa cells were transiently transfected with *SDC1*, *BGN*, or *DCN* expression vector, and treated with 50 μM of nifedipine (Nife), rifaximin (Rifa), triclabendazole (Trcl), or vitamin A (VitA) for 24 h. The cell lysates were analyzed by western blotting for the FLAG tag attached to the core protein introduced. All experiments were independently repeated three times and quantified by densitometry. The bar graph represents the average and S.D. of the three experiments. Statistical analysis was performed in comparison with the DMSO-treated sample. Statistical significance was calculated only between the DMSO treatment and the triclabendazole treatment group by Welch’s *t* test. (∗*p* < 0.05, ∗∗*p* < 0.01, ∗∗∗*p* < 0.001).

**Figure 5:**
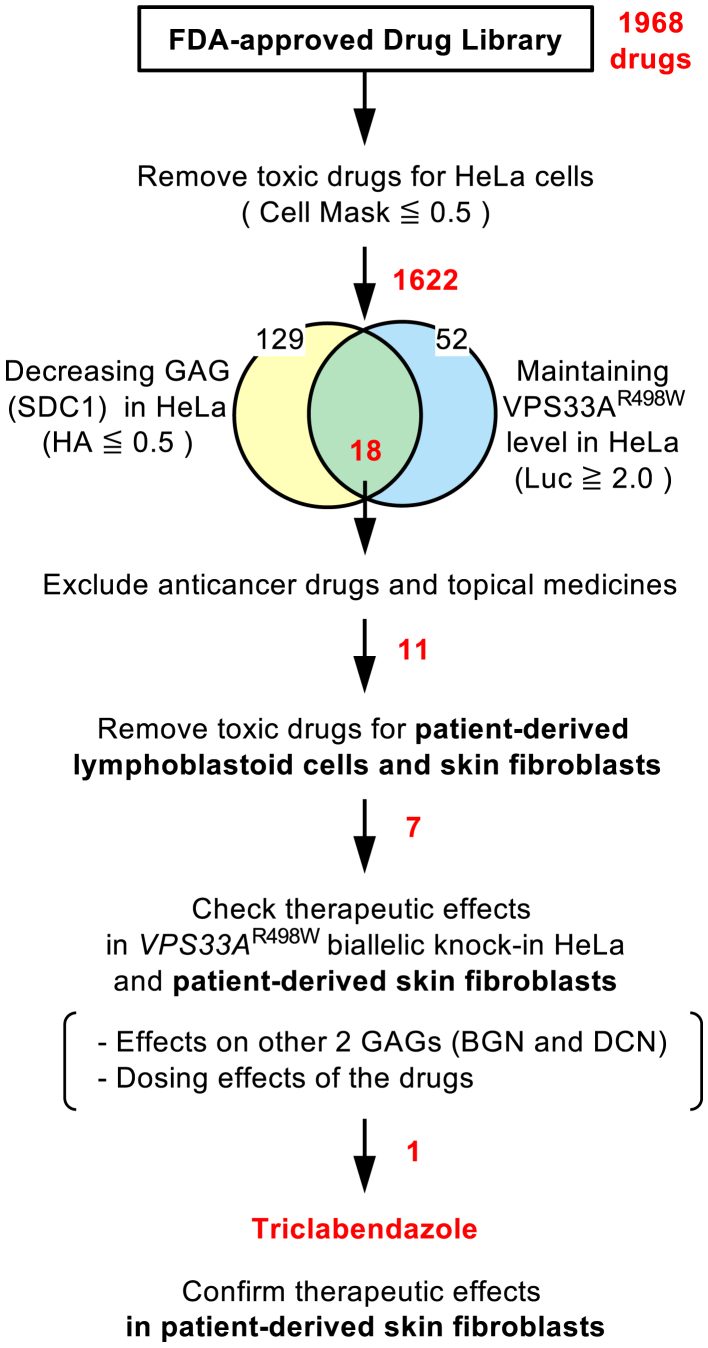
The schematic summary of the drug screening workflow The number next to the Venn diagram or arrow indicates the number of the relevant drugs. Of these, the numbers in red indicate the number of agents selected through the screenings.

It is currently unknown that the molecular mechanism, how triclabendazole could reduce intracellular GAG core protein levels. However, this effect is observed not only in *VPS33A* KI cells with the MPSPS mutation but also in WT HeLa cells and normal skin fibroblasts. This suggests that the mechanism of action of triclabendazole is not specific to the MPSPS mutation but rather directly affects the intrinsic GAG metabolic system, and that triclabendazole may apply to a wider range of mucopolysaccharide accumulation diseases, such as mucopolysaccharidoses and mucolipidoses. To test this possibility, we have generated *IDUA* KO cells (MPS Type I model), *ARSB* KO cells (MPS Type VI model), *GUSB* KO (MPS Type VII model), and *GNPTAG* KO cells (Mucolipidosis Type II/ML Type II model) by genome editing, and tested the GAG core protein suppressive effect of triclabendazole on these disease model cells. These MPS and ML model cells were transfected with tagged *SDC1*, *BGN*, or *DCN* expression vectors and treated with triclabendazole in parallel with other candidate drugs, including nifedipine, rifaximin, and vitamin A. As a result, only triclabendazole had GAG-reducing effects in all MPS and MS model cells tested (Figure 4). Other agents were effective on some model cells or some specific GAG core proteins, but their effects were limited (Figure 4). These results indicate that triclabendazole may be a potential drug with broad applicability not only for MPSPS but also for congenital metabolic diseases with GAG accumulation. Here, we summarize the overall workflow for the drug screening in this study in Figure 5.

### Triclabendazole-induced decrease in GAG core protein reflects a reduction of the accompanying glycan chain

Except for hyaluronic acid, GAGs exist with specific glycans attached to the core protein.^30^^,^^36^ Since most GAGs have specific characteristics depending on corresponding glycan chains that are responsible for most of their physiological activity,^30^ it is necessary to examine the effects of triclabendazole on glycans to elucidate therapeutic effects against the disease with mucopolysaccharide (glycan) accumulation. Since antibodies that recognize the glycan moiety are limited and expensive, we used a tagged core protein as an indirect indicator for cellular GAG levels in our screening system. triclabendazole has the effect of decreasing the level of the over-expressed core proteins, but this does not necessarily mean a decrease in the amount of glycans. We, therefore, tried several experiments to evaluate the effect of triclabendazole on GAG glycans.

**Figure 6:**
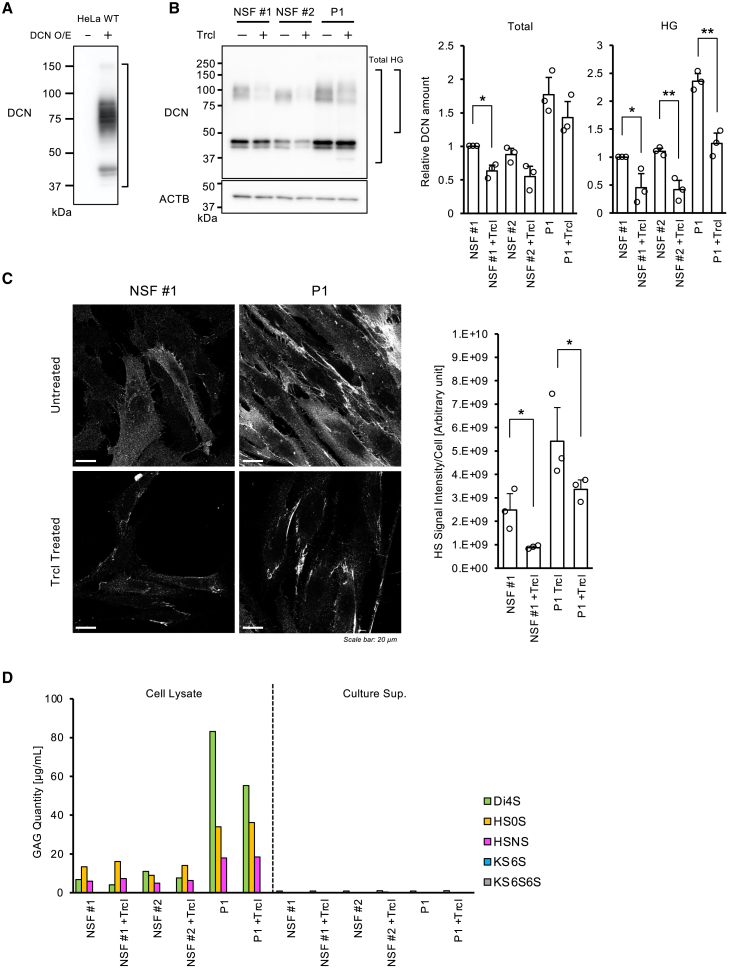
Suppressive effects of triclabendazole on the glycan levels (A) High glycosylation pattern of exogenously introduced DCN protein in HeLa cells. The DCN expression vector was transiently introduced into HeLa cells and expressed for 24 h. (B) The effects of triclabendazole on the endogenous DCN levels in normal and patient-derived skin fibroblasts. The fibroblasts were cultured in the presence or absence of the 10 μM triclabendazole for 72 h. The cell lysates were collected and analyzed for endogenous DCN by western blotting. Total and Highly Glycosylated (indicated as HG) DCN were independently quantified. Data are shown relative to the amount of DCN of untreated normal skin fibroblasts (NSF#1). Data represent the mean and S.D. of three repeated experiments. Statistical significance was calculated between the untreated and the triclabendazole treated samples by Welch’s *t* test. (∗*p* < 0.05, ∗∗*p* < 0.01). (C) Immunofluorescence analysis of Heparan sulfate (HS) in the skin fibroblasts. Normal Skin Fibroblasts (NSF #1) and the patient 1-derived skin fibroblast (P1) were treated or left untreated with 10 μM triclabendazole for 72 h. The cells were fixed and stained for HS with anti-HS-specific antibody (clone #10E4), followed by Alexa 647-labelled secondary antibody. The fluorescence images were captured by a confocal microscope. Representative images are shown for each. The fluorescence intensity of each field was divided by the number of cells (counted by DAPI staining). The bar graph represents the average and S.D. from three fields for each condition. Statistical significance was calculated between the untreated and the triclabendazole -treated samples by Welch’s *t* test. (∗*p* < 0.05). Scale bar: 10 μm. (D) Quantification of GAGs by mass spectrometry. The normal skin fibroblasts (NSF#1 and #2) and patient-derived skin fibroblasts (P1) were treated or left untreated with 10 μM of triclabendazole for 72 h. The cells were harvested by scraping after PBS washes and sonicated in PBS on ice. The inputs for the mass spectrometry were adjusted according to the sample protein concentrations.

Firstly, as shown in Figure 6A, the exogenous DCN protein in HeLa cells showed a smear blotting pattern when it was detected with an anti-Decorin (DCN) antibody. Although the blotting pattern was different from the one detected with an anti-tag antibody (Figure 3), the smear bands at a high-molecular-weight position suggest that the over-expressed core protein also has long glycan chains. Next, to examine the effect of triclabendazole on endogenous DCN, normal and patient-derived skin fibroblasts were treated with triclabendazole and subjected to western blotting using the same anti-DCN antibody. The results showed that triclabendazole tended to reduce endogenous DCN in both normal and patient-derived cells. When the total amount of DCN, including low molecular weight bands, was quantified, the effect was limited. However, by focusing on high molecular weight bands that are thought to contain a lot of glycans, a significant reduction was observed in all cells (Figure 6B). This result suggests that triclabendazole has an inhibitory effect not only on the amount of core protein (DCN) but also on the glycan moiety (i.e., dermatan sulfate, DS). In addition to western blotting, which is still an indirect method for glycan quantification, we also tried immunofluorescence analysis of the skin fibroblasts with an anti-heparan sulfate (HS)-specific antibody. As shown in Figure 6C, a massive accumulation of HS was observed in patient (P1) derived skin fibroblasts. The triclabendazole treatment significantly reduced the HS level both in the normal and patient fibroblasts, which can exclude the possibility that the GAG-reducing effects of triclabendazole are an artifact caused by the model cell screening strategy. Furthermore, we also quantified the DS, HS, and keratan sulfate (KS) by mass spectrometry (MS). The cell lysates and culture supernatants were collected from the cultures of normal (NSF #1 and #2) and the patient-derived skin fibroblasts (P1) treated with or without triclabendazole for 72 h. The MS analysis revealed the accumulation of DS (Di4S) and HS (HS0S and HSNS) in the cell lysates from patient skin fibroblasts, as it was reported. Importantly, the amount of DS was reduced by the treatment of the cells with triclabendazole, which also provides strong evidence that this drug can suppress the GAGs, including both core proteins and glycans.

### Triclabendazole preferentially suppresses the transcription of GAG core proteins rather than enhancing their degradation

Triclabendazole was originally developed as a therapeutic agent for the treatment of fascioliasis in humans and livestock.^37^ It is thought to act against the liver parasite via inhibition of microtubule polymerization,^38^^,^^39^ and the drug probably penetrates biological membranes by diffusion and exerts toxicity to fluke cells. On the other hand, no clear cytotoxicity or teratogenicity has been reported in animal studies using mammals such as mice and rats, or in human studies. Therefore, the molecular mechanism of the GAG-reducing effect of triclabendazole revealed in this study is completely unknown. We, thus, investigated whether the inhibitory effect of triclabendazole is due to inhibition of GAG biosynthesis or enhancement of its degradation.

**Figure 7:**
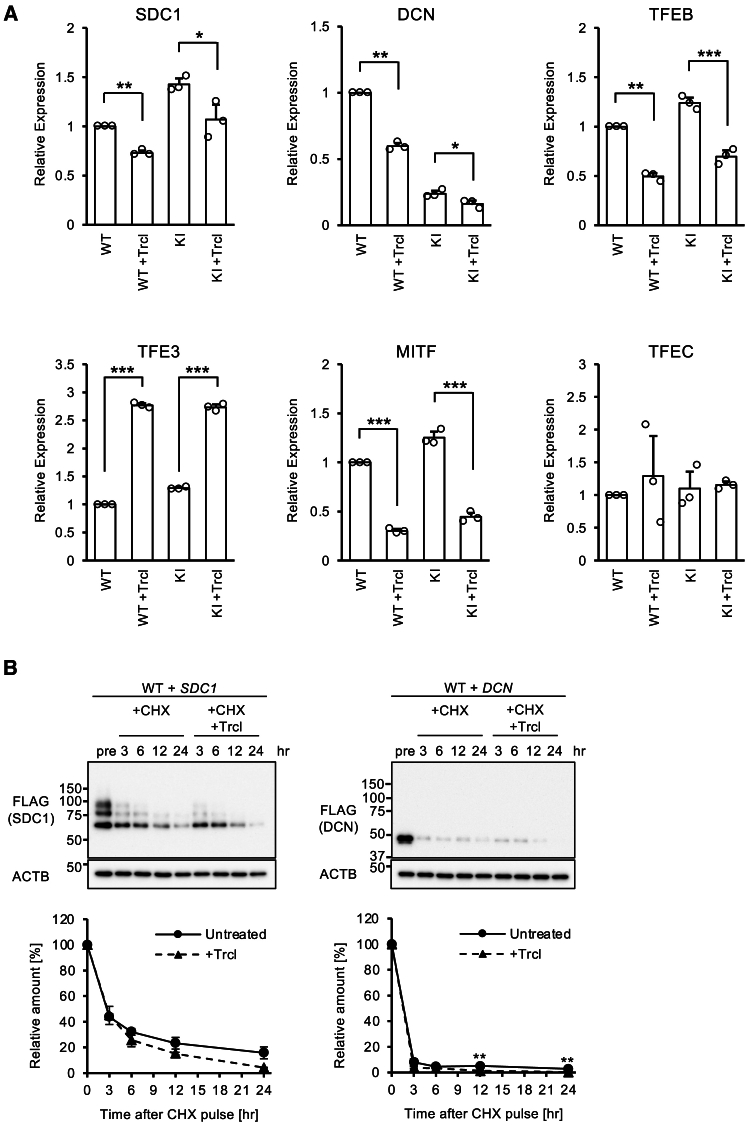
Triclabendazole suppresses the GAG core proteins at the transcriptional level (A) Quantification of GAG-related and unrelated genes’ transcripts by real-time RT-PCR. WT and the *VPS33A* KI cells were treated or left untreated with 50 μM of triclabendazole for 24 h. Total RNA from these samples was extracted and subjected to reverse transcription with random hexamer. Real-time PCR was performed using SYBR Green and specific primer sets for each gene, and quantification was performed from the amplification curves. A standard curve was drawn for each target gene using a serially diluted untreated WT HeLa sample, and the relative values of the other samples were calculated from the curve. The bar graph represents the mean and S.D. of three independent experiments. The target gene name was indicated at the top of each graph. Statistical significance was calculated between the untreated and the triclabendazole -treated samples by Welch’s *t* test. (∗*p* < 0.05, ∗∗*p* < 0.01, ∗∗∗*p* < 0.001). (B) Effect of triclabendazole on the degradation rate of GAG core protein. HeLa cells were transiently transfected with FLAG-tagged *SDC1* or *DCN* expression vector and expressed for 24 h. 50 μg/mL of CHX was added to these cells to suppress the synthesis of the *de novo* GAG core protein, and further cultured in the absence or presence of 50 μM triclabendazole. The cells were collected at the time points indicated, and the time course of degradation was monitored by Western blotting. The sample before the addition of CHX (pre) was set at 100%, and the amount of residual core protein is shown as a relative value. The data point on the line graph represents the mean and S.D. of the three independent experiments. Statistical analyses were performed by comparing the samples with and without triclabendazole treatment at the same time points using Student’s *t* test. (∗∗*p* < 0.01).

First, cDNA was prepared from triclabendazole -treated and untreated WT and *VPS33A* KI HeLa cells, and quantitative RT-PCR was performed to examine the effect of triclabendazole on the transcription of endogenously expressed *SDC1* and *DCN* genes. The results showed that triclabendazole significantly suppressed the transcription of these two core proteins (Figure 7A). To exclude the possibility that triclabendazole suppresses the global transcription of the cells, we similarly examined the transcription of four genes belonging to the *TFEB* family, which are not directly related to GAG production. As a result, the transcription of *TFEB* and *MITF* genes was suppressed, while that of *TFE3* was rather enhanced by triclabendazole, and that of *TFEC* was not affected (Figure 7A). These results raise the possibility that triclabendazole may selectively suppress the transcription of GAG core proteins rather than in a non-specific manner.

Finally, we investigated the effect of triclabendazole on the degradation of GAG core proteins using the CHX Pulse-Chase assay. As in the previous experiments, HeLa cells were transfected with tagged *SDC1* or *DCN*, and their translation was inhibited by the treatment with CHX, then the degradation time course of each core protein in the absence or presence of triclabendazole was followed up to 24 h. As a result, only minimal statistically significant differences were observed, although triclabendazole treatment tended to slightly hasten the GAG core protein degradation (Figure 7B). Although these results are not sufficient to conclude, it is conceivable that the inhibitory effect of triclabendazole on cellular GAG levels could be suppression of GAG biosynthesis at the transcriptional level rather than an acceleration of GAG degradation.

In this study, we found that although the MPSPS mutation markedly impairs the stability of the VPS33A protein, its function per molecule is comparable to that of the wild type as far as autophagy activity is concerned. On the other hand, this means that the molecular mechanism of how the VPS33A mutation leads to MPSPS remains unknown. To circumvent this problem, we screened the FDA-approved drugs that reduce GAG levels in cells and identified triclabendazole as a very promising candidate. Triclabendazole showed significant therapeutic effects not only in cell-based *in vitro* experiments but also in MPSPS model mice, though the effects were limited. Although the molecular mechanism of the GAG suppression remains to be clarified in more detail, it is expected that treatment with triclabendazole will apply not only to MPSPS but also to a wide range of related diseases associated with GAG accumulation.

## Discussion

The causative mutation in MPSPS results in the single amino acid substitution (p.R498W) in VPS33A, one subunit of the tethering complex. Our results showed that this mutation impairs the stability of the VPS33A protein, resulting in a marked decrease in the amount of VPS33A in cells. Despite this, autophagy was normal in cells harboring the mutant VPS33A. The gene silencing experiments indicate that only a minimal amount (less than 10% of WT cells) of VPS33A is required to drive autophagy (Figure S1E). To accurately assess the impact of reduced stability of VPS33A on autophagy, an experimental system was needed to precisely evaluate the correlation between protein expression levels and function.

DEFAC, which we have developed, is an excellent method for assessing the relationship between target protein levels and focused functions and can be optimized and applied in a large variety of experiments. Simultaneous measurement of the expression levels of target molecules and the amount of functional probes in individual cells can capture the function in a categorical group with a certain level of target molecules. In the present study, we applied the LC3 flux assay,^20^ commonly used in autophagy studies, to DEFAC. This experimental approach revealed that the VPS33A^p.R498W^ protein, no matter how low its level is, retains an almost comparable function in driving autophagy to the WT VPS33A (Figures 1B and 1D).

Generally, in studies that examine the impact of protein level on cellular function, the amount of protein produced from exogenously introduced genes varies from cell to cell, even within a uniform genetic background. In such heterogeneous cell populations, there may be a mixture of cells with enhanced or reduced function depending on the amount of protein produced in each cell. Generic methods, such as western blotting, that analyze the integrated amount of protein and function of whole populations, are difficult to apply to the study of exquisite cellular responses. The concept of DEFAC is a method like the “propensity score matching” strategy in statistics. It can compare the function of a population with equivalent protein levels (i.e., expression background) and is not influenced by the efficiency of transfection/expression of the exogenous gene to be compared. It allows a purely qualitative functional evaluation of a target molecule.

In this study, we have classified the cell populations into five groups according to the GFP intensity (i.e., VPS33A level), but it is also possible to re-analyze the results by grouping them more finely, even after the experiment or analysis is finished. In this respect, it also has another advantage over the drug-induced expression systems or regulatory systems with protein degrons. DEFAC can be applied to any system in which both the amount of the target molecule whose function is to be checked, and the reporter reflecting that function, if they have a different color of fluorescence. Furthermore, by using fluorescent dyes of different excitation or emission wavelengths as reporters of the cellular functions to be measured, multiple cellular functions can also be monitored in a single cell simultaneously. There are unlimited possibilities for application as the target protein and functional probes can be designed freely.

Unlike classical MPS, MPSPS is not a disease caused by a deficiency of lysosomal enzymes.^1^ The known function of *VPS33A*, the gene responsible for MPSPS, is to regulate membrane trafficking within autophagy and endocytosis.^13^^,^^16^ At this point, however, impairment of endocytosis or autophagy is not reported in the cells with the MPSPS mutation. In fact, in our study, the MPSPS mutant of VPS33A was shown to retain the equivalent function of the WT protein as far as autophagy activity is concerned (Figure 1). On the other hand, dysfunction in the lysosomal system is strongly suggested by the similar clinical phenotype of MPSPS to lysosomal storage diseases and abnormal accumulation of endolysosomes in patient skin fibroblasts^23^^,^^40^^,^^41^ (Figure 3C). The highly similar clinical manifestations of MPSPS and MPS can be attributed to the adverse effects of the excessive accumulation of GAGs, which may disturb the endosome systems in both types of disease. From these facts, it can be inferred that MPSPS might be caused by a defect in the intracellular uptake and/or transport of GAGs into lysosomes.^42^

The clinical similarities between MPS and MPSPS, both caused by defects in a series of GAG metabolism pathways, are interesting. On the other hand, MPSPS patients have been reported to have hematopoietic disorders and renal failure,^5^ which are not generally observed in MPS patients, and it is reasonable to assume that these symptoms are caused by factors other than GAG accumulation. Thus, the p.R498W mutation in VPS33A may affect the unknown function of VPS33A apart from the HOPS or CORVET. Our previous study has shown that receptor-mediated endocytosis and macropinocytosis are differentially regulated by the combination of the conventional and novel-type tethering complexes, including VPS33A.^17^^,^^43^ These novel tethering complexes may be involved in the unknown functions of VPS33A, or VPS33A may have further additional functions unrelated to them. The pathogenesis of MPSPS shall be established by aberrant membrane trafficking through the reduced abundance of VPS33A and/or by a specific function yet to be known of the VPS33A p.R498W variant on GAG metabolism. It still remains possible that some specific organs are affected by the reduction of the unstabilized protein with its severe thresholds of VPS33A amount for autophagy or endocytosis. Thus, further studies are needed to elucidate the full extent of the effects of the p.R498W variation of VPS33A on membrane trafficking and GAG metabolism.

The drugs approved by the FDA have already passed clinical trials and have certain safety and pharmacokinetic profiles. Therefore, the application of these drugs to other diseases “drug repositioning” is a very effective means in that the process of drug approval in different countries can be largely omitted, and clinical application can proceed rapidly.^44^ Here, triclabendazole was identified as a therapeutic candidate against MPSPS. Triclabendazole effectively suppressed the exogenously introduced GAG core proteins, including SDC1, BGN, and DCN, both in the MPSPS patient skin fibroblasts and the model cells (Figures 3A and 3B). Triclabendazole reduced not only core proteins produced by forced expression but also endogenous DCN in dermal fibroblasts from healthy individuals and MPSPS patients (Figure 6B). Therefore, the GAG-inhibitory effects of triclabendazole cannot be considered as an artifact produced in the model cells. Many of the physiological activities of GAGs, such as water retention and binding to growth factors, are dependent on their glycan chains.^30^^,^^36^ Although triclabendazole was selected by screening FDA-approved drugs using the SDC1 as an indicator for GAG amount, the amount of glycan chains was found to be decreased by this drug along with its core protein (Figures 6B–6D). Therefore, it is expected that triclabendazole can reduce excess amounts of GAGs, which include disease-causing glycans, in MPSPS patients. Electron micrographs showed abnormal endolysosomal accumulation in patient fibroblasts. This suggests that excessive intracellular accumulation of GAGs may cause abnormalities in the endolysosomal system. These abnormal endolysosomes were significantly reduced in triclabendazole -treated cells (Figure 6C), suggesting triclabendazole can “cure” endosomal abnormalities by reducing GAG load within the endosome system.

Among the drugs that had been selected in the screening, the inhibitory effect of triclabendazole on GAGs *in vitro* cultured cell experiments was outstanding. These results prompted us to investigate the therapeutic effect of triclabendazole *in vivo* using mouse models. We have established the *Vps33a*^*p.R500W*^ KI mouse, whose mutation corresponds to the human MPSPS variation. Different from the human cases, the homozygote mice for the mutant KI allele were embryonic lethal, and could not be delivered as live neonates. Thus, these KI mice are not a perfect MPSPS model, but we utilized them to evaluate the therapeutic effects of triclabendazole *in vivo*. Triclabendazole administered to mother mice via drinking water did not result in live birth of KI homozygotes, but viable fetuses *in utero* just before delivery were separated according to Mendelian ratios (Figure 3D). This suggests that triclabendazole acted transplacentally on the fetus, reducing the lethal effects of GAG accumulation during embryogenesis. Curiously, even in the untreated group of mice, very rare individuals could be found that survived until just before birth (Figure 3D). There is one possibility that GAG accumulation may bring a crisis in the mouse fetus at a very early stage of gestation, but if that phase is overcome by chance, untreated fetuses also could survive to some extent, similarly to triclabendazole -treated ones. It has been reported that a human fetus with MPSPS also represented abnormalities during the late stage of pregnancy.^7^ The results of this animal study provide the possibility that the administration of triclabendazole can be an early treatment option for MPSPS patients.

Triclabendazole is a class of benzimidazole antiparasitic drugs applied to fascioliasis in humans and livestock.^37^^,^^38^^,^^39^^,^^45^ This drug is administered orally, absorbed through the gastrointestinal tract, then transferred into the blood, and is thought to exert its effect on parasites in the liver through microtubule assembly inhibition.^37^^,^^39^ No clear toxicity or teratogenicity, however, has been reported in mammalian animals such as mice and rats, livestock, or humans in the range of normal use.^38^^,^^46^^,^^47^ In fact, in our mouse experiments, WT and the heterozygous KI mice were delivered from the triclabendazole -treated mothers without problems. Furthermore, even though they continued to receive triclabendazole via maternal milk or drinking water until weaning, there were no apparent abnormalities observed, at least in appearance, and no problems with reproduction for the next generation. Although triclabendazole is essentially a short-term drug administered for deworming. But these results indicate that it is possible to apply triclabendazole to pregnant women carrying MPSPS fetuses and newborns for the long term. It will be necessary to confirm the efficacy of the treatment or adverse effects *in vivo* extensively using animal models in the future.

Triclabendazole suppressed GAGs not only in MPSPS cells but also in WT HeLa cells or normal skin fibroblasts (Figures 3B, 6B, 6C, and S7). Thus, the drug does not seem to act on the MPSPS mutant VPS33A protein in a specific manner but may act directly on the GAG metabolic pathway and can potentially expand its applications to other diseases than the ultra-rare MPSPS. Indeed, triclabendazole reduced the transduced GAG core proteins in the MPS model cells, including *IDUA* KO cells, *ARSB* KO cells, and *GUSB* KO cells, as well as in *GNPTAB* KO cells, a model cell for mucolipidosis type II. Enzyme replacement therapy (ERT) is known as the standard treatment for MPS, while stem-cell transplantation and gene therapy are being developed as advanced alternative treatments,^48^ but triclabendazole could be a new treatment option next to these therapies. In addition to p.R498W, the p.R200P variant in the *VPS33A* gene has recently been reported to cause a juvenile disease.^49^^,^^50^ Because the domains of the pathogenic mutations in VPS33A are far from each other (p.R200P in the domain D2-1 and pR498W in the domain D2-2, Figure 1A), it is still debatable whether this disease has the same pathomechanisms as MPSPS. Nevertheless, the patients with p.R200P also excrete excessive GAG in their urine,^49^^,^^50^ and GAG likely accumulates in the body of this patient. In summary, triclabendazole would be a highly potent therapeutic candidate not only for MPSPS but also for GAG-accumulating related diseases.

Heparan sulfate with SDC1 as a core protein, which was also found to be reduced in the present study, is recognized as a receptor for SARS-CoV-2 and bacterial entry.^51^^,^^52^ Heparan sulfate acts on signal transduction of growth factors such as HGF, FGF, PDGF, and VEGF pathways and is involved in cell proliferation and angiogenesis.^53^ The regulation of GAGs has a clinical impact on a wide range of diseases. Triclabendazole has the potential to modulate the pathophysiology of not only hereditary disorders of mucopolysaccharide metabolism seen in MPSPS, MPS, and ML, but also infectious diseases and cancer.

Our results demonstrate that triclabendazole has promising therapeutic effects at the cellular and *in vivo* level, the molecular mechanism of how triclabendazole reduces GAGs remains to be elucidated. Although it was limited, our results showed that triclabendazole suppressed the expression of the core protein genes at least at the transcriptional level (Figure 7A). Because most of the GAG glycans are attached to core proteins, it is expected that the amount of glycans will decrease when the production of core proteins is suppressed. In addition, triclabendazole showed a preferentially higher inhibitory effect on the highly glycosylated DCN (Figure 6B), and the degradation of GAGs was slightly hastened in the presence of this drug (Figure 7B). These data suggest that triclabendazole may also affect the glycosylation and degradation processes of the GAG core protein. Further investigation of how triclabendazole acts on GAG metabolism and how it modulates MPSPS pathophysiology *in vivo* will open new avenues for the treatment of a broad group of diseases associated with abnormal mucopolysaccharide metabolism and membrane trafficking. Furthermore, the effects of abnormalities in the tethering complex on living organisms have been successively revealed at the *in vivo* level.^16^ Our research sheds light on them and is to facilitate our understanding of the related diseases,^54^^,^^55^ as well.

### Limitations of the study

The major limitations of the MPSPS study are the regional localization and the ultra-rare frequency of patients. Only ∼20 cases have been reported worldwide, and most of them were from the Republic of Sakha. Due to its infrequency and the fact that it was recognized in 2017, the clinical knowledge of MPSPS regarding etiology and patient samples available is limited. We established a research collaboration between institutes in Japan and the Republic of Sakha, and are collecting patient information and materials. The lack of an appropriate model animal in the current situation is also another limitation. We have generated KI mice in which the mutation corresponding to the human MPSPS was introduced into the mouse Vps33a gene, but unlike the human case, the mice were embryonic lethal. Therefore, it is impossible to analyze the pathogenesis of MPSPS or evaluate the effects of drug treatment after birth using this KI mouse. A fundamental understanding of MPSPS will help overcome the problems described above in MPSPS research, and the current study is a worthy step in that direction.

## Resource availability

### Lead contact

Further information and requests for resources and reagents should be directed to and will be fulfilled by the lead contact, Takanobu Otomo (otomo@med.kawasaki-m.ac.jp).

### Materials availability

The materials generated in the current study are not publicly available but are available from the lead contact author upon reasonable request.

### Data and code availability

• Data: All data are available in the main text or the supplemental information. All data (including raw data of drug screening used to produce a survivability histogram, a scatterplot of stability/effectiveness, and a screening flow chart) reported in this paper will be shared by the lead contact author upon request.
• Code: This paper does not include any original code.
• Additional information: Any additional information required to reanalyze the data reported in this paper is available from the lead contact author upon request.

## Acknowledgments

This research was supported by 10.13039/501100001691JSPS KAKENHI grants (JP17H05088, JP18F18094, and JP22H03046), 10.13039/100009619AMED grant (JP24ym0126810 via Okayama University), 10.13039/100016923Hoansha Foundation, and our institutional research grant (R03B-007) to T.O., 10.13039/100016189Wesco Scientific Promotion Foundation to S.T., and 10.13039/501100003443Ministry of Education and Science of the Russian Federation (FSRG-2020-0014) to N.M. We would like to express our gratitude to Dr. Torayuki Okuyama for his cooperation in the mass spectrometry of glycosaminoglycans and to Mr. Takeyuki Akiyama, Honorary Chairman of the Japan Mucopolysaccharidosis Patient Family Group, for his supervision. We also thank Mr. Nobuaki Matsuda from the Bioimaging Research Unit for the electron micrograph, Ms. Mariko Inoue from the Medical Bioresource Research Unit for the generation of *Vps33a* KI mice in our school, and Dr. Yukiko Kawakami and Ms. Saki Fujita for their technical assistance in our lab.

## Author contributions

T.O. designed and supervised research; S.T., F.V., V.S., M.T., Y.M., R.I., T.M., and T.F. performed research and analyzed data; S.T. and T.O. wrote the manuscript. All authors reviewed, discussed, and edited the manuscript.

## Declaration of interests

The authors declare a potential conflict of interest. triclabendazole, used in this study, is the subject of a pending patent application (patent application JP2025-011019) concerning its intended therapeutic use.

## STAR★Methods

### Key resources table

**Table:** 

REAGENT or RESOURCE	SOURCE	IDENTIFIER
**Antibodies**		

Rabbit polyclonal anti-LC3	MBL	Cat#PM036; RRID: AB_2274121
Rabbit polyclonal anti-p62(SQSTM1)	MBL	Cat#PM045; RRID: AB_1279301
Rabbit polyclonal anti-NDP52	GeneTex	Cat#GTX115378; RRID: AB_10620266
Rabbit polyclonal anti-VPS33A	Novus	Cat#NBP2-20872; RRID: AB_3265436
Mouse monoclonal anti-Decorin (clone #115402)	R&D	Cat#MAB143; RRID: AB_2230373
Mouse monoclonal anti-Myc (clone #My3)	MBL	Cat#M192-3; RRID: AB_11160947
Mouse monoclonal anti-FLAG (clone #M2)	Merck (Sigma-Aldrich)	Cat#F3165; RRID: AB_259529
Mouse monoclonal anti-HA (clone #F-7)	Santa Cruz	Cat#sc-7392; RRID: AB_627809
Mouse monoclonal anti-DYKDDDK (FLAG)-HRP conjugated (clone #1E6)	FujiFilm-Wako	Cat#015-22391; RRID: AB_2877162
Mouse monoclonal anti-Heparan Sulfate (clone #10E4)	AMSBIO	Cat#370255; RRID: AB_10891554

**Bacterial and virus strains**		

pMXsIP-GFP-VPS33A	Jiang P. et al.^56^	Addgene: 67022
pMXsIP-HA-SDC1-FLAG	This paper	N/A
pMXsIP-HA-BGN-FLAG	This paper	N/A

**Chemicals, peptides, and recombinant proteins**		

DiscoveryProbe™ FDA-approved Drug Library	APExBIO	Cat#L1021
Acitretin	TCI	Cat#A3097
Andrographolide	TCI	Cat#A2459
Apigenin	FujiFilm-Wako	Cat#012-18913
Berberine Sulfate	TCI	Cat#B0451
Dimethyl Fumarate	TCI	Cat#F0069
Entacapone	TCI	Cat#E0961
Harmine hydrochloride	TCI	Cat#H0002
Nifedipine	FujiFilm-Wako	Cat#145-05781
Rifaximin	TCI	Cat#R0101
Triclabendazole	TCI	Cat#T2826
Vitamin A	FujiFilm-Wako	Cat#20241
Bafilomycin A1	Cayman Chemical	Cat# 11038
Cycloheximide	Nacalai-Tesque	Cat# 06741-91
Chondroitinase B	Iduron	Cat#BN7
Heparinase I/III	Sigma-Aldrich	Cat#H3917
Keratanase II	GlycoSyn	Cat#FC-156
Chondrosine monohydrate	BIOSYNTH	Cat#OA10113
Dermatan Sulfate	TCI	Cat#D3672
Heparan Sulfate	Iduron	Cat#BN1GAG-HS01
Keratan Sulfate	PG Research	Cat# NaKS2(PNC)3

**Critical commercial assays**		

Human heparan sulfate, HS ELISA Kit	CUSABIO	Cat#CSB-E09585h

**Experimental models: Cell lines**		

HeLa Kyoto	Landry J. et al.^35^	N/A
Normal Skin Fibroblast (as NFS#1 in this paper)	Thermo Fisher (Gibco)	Cat#C0135C
Normal Skin Fibroblast (as NFS#2 in this paper)	Lonza	Cat#CC-2511
Normal Skin Fibroblast (as NFS#3 in this paper)	Kurabo	Cat#KF-4109
MPSPS Patient-derived Skin Fibroblast (as P1 in this paper)	Kondo H. et al.^1^	N/A
MPSPS Patient-derived Skin Fibroblast (as P2 in this paper)	Kondo H. et al.^1^	N/A
Healthy Donor-derived LCL	This paper	N/A
MPSPS Patient-derived LCL	This paper	N/A
MPSPS Carrier-derived LCL	This paper	N/A

**Experimental models: Organisms/strains**		

Mouse: C57BL/6JmsSlc	SLC Japan	N/A
Mouse: *Vps33a*^*p.R500W*^ KI/C57BL/6J	This paper	N/A

**Oligonucleotides**		

Human *VPS33A* KO Target sequence: 5’ CATAT TGACC TTACA CAACC 3’	This paper	N/A
Human *VPS33A* KI (p.R498W) Target sequence: 5’ TGCCC CGCTC AGTGT GCGGC 3’	This paper	N/A
Human *VPS33A* KI (p.R498W) Donor Oligo: 5’ CAGAA CCCCA CGGAC ATATC GTATG TGTAC AGTGG GTATG CCCCA CTCAG TGTGT GGCTT GCCCA GCTGC TTTCC CGGCC TGGCT GGCGG AGCAT CGAGG 3’	This paper	N/A
Mouse *Vps33a* KI (p.R500W) Target sequence: 5' AGCAT AACCG CTGTA CACAT 3' (anti-sense)	This paper	N/A
Mouse *Vps33a* KI (p.R500W) Donor Oligo: 5’ CAGAA CCCCA CGGAC ATATC GTATG TGTAC AGTGG GTATG CCCCA CTCAG TGTGT GGCTT GCCCA GCTGC TTTCC CGGCC TGGCT GGCGG AGCAT CGAGG 3’	This paper	N/A
Quantitative RT-PCR primer for SDC1 (Fwd): 5’ TGGGG ATGAC TCTGA CAACT TC 3’	This paper	N/A
Quantitative RT-PCR primer for SDC1 (Rev): 5’ TGCGT GTCCT TCCAA GTGG 3’	This paper	N/A
Quantitative RT-PCR primer for DCN (Fwd): 5’ AAGAT GAGGC TTCTG GGATA GG 3’	This paper	N/A
Quantitative RT-PCR primer for DCN (Rev): 5’ TCGAA GATGG CATTG ACAGC 3’	This paper	N/A
Quantitative RT-PCR primer for TFEB (Fwd): 5’ GAGCG GCAGA AGAAA GACAA TC 3’	This paper	N/A
Quantitative RT-PCR primer for TFEB (Rev): 5’ GATCA GCATT CCCAA CTCCT TG 3’	This paper	N/A
Quantitative RT-PCR primer for TFE3 (Fwd): 5’ TGATC CTGAC AGCTT CTACG AG 3’	This paper	N/A
Quantitative RT-PCR primer for TFE3 (Rev): 5’ ACGAT GCAGA GAGTG TAGCT G 3’	This paper	N/A
Quantitative RT-PCR primer for MITF (Fwd): 5’ GGCTA TGCTT ACGCT TAACT CC 3’	This paper	N/A
Quantitative RT-PCR primer for MITF (Rev): 5’ ACGCT CGTGA ATGTG TGTTC 3’	This paper	N/A
Quantitative RT-PCR primer for TFEC (Fwd): 5’ TGGCA CGGTT GATTT AGGTG 3’	This paper	N/A
Quantitative RT-PCR primer for TFEC (Rev): 5’ AGACA CAGTC AGTTG TTGGC 3’	This paper	N/A
Quantitative RT-PCR primer for GAPDH (Fwd): 5’ CAATG ACCCC TTCAT TGACC 3’	This paper	N/A
Quantitative RT-PCR primer for GAPDH (Rev): 5’ GACAA GCTTC CCGTT CTCAG 3’	This paper	N/A

**Software and algorithms**		

Image Lab Ver. 6.1.0	Bio-Rad	N/A
Fiji (ImageJ2) Ver. 2.14.0/1.54f	Schindelin J. et al.^57^	http://imagej.net/
EZR Ver. 1.54	Kanda Y.^58^	https://www.jichi.ac.jp/usr/hema/EZR/statmed.html
ZEN Ver. 3.10	Carl-Zeiss	https://www.zeiss.com/microscopy/en/products/software/zeiss-zen.html
Design and Analysis Software v1.5.1, QuantStudio 3 and 5 systems	Thermo Fisher	https://www.thermofisher.com/jp/ja/home/technical-resources/software-downloads/quantstudio-3-5-real-time-pcr-systems.html
LabSolutions LCMS Ver. 5.113	SHIMADZU	https://www.an.shimadzu.co.jp/service-support/class/download/lcms/index.html

### Experimental model and study participant details

Skin fibroblasts (N=2) and lymphoblastoid cell lines (N=1) derived from MPSPS patients were obtained from the remainder of the human material obtained during diagnosis after the consent of the patients. HeLa Kyoto cells were originally established at Kyoto University based on canonical HeLa cells and were provided to EMBL, and are now distributed worldwide without any limitation (RRID: CVCL_1922). *Vps33A*^*p.R500W*^ KI mouse was established in our animal facility using wild-type C57BL/6J mice from Japan SLC Inc. Mycoplasma infection of the cell lines is regularly confirmed to be negative by a PCR-based method.

### Method details

#### Study design

This study was conducted to elucidate the molecular pathogenesis of MPSPS, an ultra-rare disease, and to search for specific treatments (or therapeutic agents) accordingly. In this study, we attempted to elucidate the pathogenesis of MPSPS in an *in vitro* experimental system by combining these patient samples and disease model cells generated from cultured cell lines by genome editing. We further applied the model cells to generate a reporter system that uses cellular GAG and VPS33A levels as indicators and established a high-throughput screening system to search for GAG-suppressing drugs from a library of approximately 2,000 FDA-approved drugs. Large-scale screening was performed using the reporter cells, and the drug effects on cellular GAG were verified using the patient-derived cells. The first stage of screening using reporter cells identified 18 drugs that reduced GAG levels but were less cytotoxic and did not impair the stability of VPS33A. The second screening stage excluded anticancer agents, topical agents, and agents with high toxicity to patient-derived fibroblasts or lymphoblastoid cell lines, given that the agents are administered to pregnant women or newborns. Of the seven remaining drugs, we tested their effects on the patient fibroblasts for multiple GAGs, different from the GAGs used as the first screening index to identify medicines that reduce GAGs. To test the therapeutic efficacy of Triclabendazole *in vivo*, KI mice with an equivalent mutation in the *Vps33a* gene to human MPSPS were created as a disease model. Since the homozygotes were embryonic lethal, we decided to examine the life-prolonging effect of the drug on the homozygote fetuses. The heterozygous male and female mice were mated, and the pregnant mice were treated with Triclabendazole via drinking water. The fetuses from 6 mother mice for each treated and untreated group were collected at ∼18 d.p.c., and the results of genotyping were pooled and statistically processed. Except for the large-scale screening system, electron microscopy, and mass spectrometry for the measurement of glycosaminoglycans, all experiments were performed at least three times independently and reflected in the data. This is a non-invasive, non-interventional study using the remainder of the human material obtained during diagnosis. The study was approved by the ethics committee of Kawasaki Medical School (Approval Number: 5541). Consent for the use of patient specimens in this study has been obtained in an opt-out manner in accordance with the operation of Japanese ethical guidelines.

#### Cell culture

HeLa Kyoto cells (Simply described as HeLa cells in this manuscript) and the patient-derived skin fibroblasts were cultured in Dulbecco’s Modified Eagle Medium (DMEM) (Merck, MA) supplemented with 10% fetal bovine serum (FBS) and 100 units/mL penicillin, 100 μg/mL streptomycin (Nacalai-Tesque, Japan). The patient-derived lymphoblast cell lines were cultured in RPMI1640 (Merck) with 10% FBS and antibiotics. All cells were maintained in a humidified incubator with 5% CO2, 37°C. Depending on the vector constructions, 1 μg/mL of Puromycin or 50 μg/mL of Hygromycin (Nacalai-Tesque) was used to select virally gene-transfected cells.

The normal skin fibroblasts (NSF) #1, #2, and #3 were purchased from Gibco™ (Thermo Fisher, MA), Lonza (Switzerland), and Kurabo (Japan), respectively. The patient's fibroblasts were prepared as specimens for the clinical examination, and the residual cells were used in this study. Approximately 5 mm squares of buttock skin collected by punching were shredded with a clean scalpel and cultured in AmnioMAX-II™ medium (Thermo Fisher). After colonies had expanded, they were dispersed by trypsin-EDTA digestion and thereafter cultured in normal DMEM medium.

The human lymphoblastoid cell lines were established by an outsourced service. Briefly, Peripheral blood mononuclear cells were isolated from one healthy volunteer, one MPSPS patient, and her mother (carrier) by Ficoll-Paque density gradient centrifugation. The cells were infected with EB virus (B95-8 culture supernatant) and cultured in 20% FBS/RPMI in the presence of 500 ng/mL of Cyclosporin A.^59^ After stable growth began, the cells were cultured in normal medium as described above.

#### Drug library

The FDA-approved drug library was purchased from APExBIO (MA). The drugs were dissolved in DMSO and then used at 50 μM unless specifically described. The drug information and raw screening data are provided as supplemental information (Data S1).

#### Plasmids

pMXsIP GFP-VPS33A retroviral vector was a kind gift from Dr. Noboru Mizushima (Addgene plasmid # 67022; http://n2t.net/addgene:67022; RRID: Addgene 67022).^56^ The GFP-tagged VPS33AR498W MPSPS pathogenic mutant and the domain 3 deletion (Large Deletion; LD) mutant constructs were prepared by PCR-based methods using the WT plasmid as a template. *Myc-VPS41*, *Myc-VPS8*, *His-VPS18*, *FLAG-VPS11*, and *HA-VPS3* in the pcDNA3.1 expression vector used in the IP experiment were described in our previous report. The *SDC1* cDNA was obtained by RT-PCR from HeLa total RNA, and then HA and FLAG tags were added to the N-terminus and C-terminus of *SDC1* by PCR, respectively, and then subcloned into pMXsIP or pcDNA3.1 plasmid. The mutant *VPS33A* cDNA was incorporated into the pNLF1 vector (Promega, WI) to fuse with NLuc moiety.

#### Gene expression with viral transduction

Plat-E cells were transfected with the retroviral vectors in combination with pCAG-VSVG plasmid to produce pantropic pseudovirus using PEI max (Polysciences, PA). The supernatant of the transfected cells was harvested at 48 hr. and 72 hr. after the plasmids transfection and then was cleared by passing through an 8 μm-pore filter (Sartorius, Germany). The HeLa-derivative cells or the skin fibroblasts were infected with 20% or 50% of the virus supernatant with 10 μg/mL of Polybrene® (Santa Cruz Biotechnology, TX) for 48 hr., respectively.

#### Genome editing by CRISPR/Cas9

Guide sequences for gene targeting were designed using the Benchling CRISPR Guide RNA Design Tool (https://www.benchling.com/crispr/). The guide sequence for VPS33A knock-out and *VPS33A* p.R498W knock-in is 5’ CATAT TGACC TTACA CAACC 3’, 5’ TGCCC CGCTC AGTGT GCGGC 3’, respectively. Donor oligo sequence for homologous recombination to knock in p.R498W into *VPS33A* gene is 5’ CAGAA CCCCA CGGAC ATATC GTATG TGTAC AGTGG GTATG CCCCA CTCAG TGTGT GGCTT GCCCA GCTGC TTTCC CGGCC TGGCT GGCGG AGCAT CGAGG 3’. Double-stranded DNA coding guide RNA sequences were prepared by annealing synthesized complementary DNA oligo pairs on a thermal cycler and then cloned into the pSpCas9(BB)-2A-GFP (pX458) vector (a gift from Feng Zhang (Addgene plasmid # 48138; http://n2t.net/addgene:48138; RRID: Addgene 48138)).^60^ The targeting plasmid was transfected into HeLa Kyoto cells with Effectene Transfection reagent (Qiagen, Germany). After 48 hr. of transfection, GFP^+^ populations were sorted into single-cell cultures in 96-well plates by FACSAria III™ Cell Sorter (BD Biosciences, CA). Genomic DNA from each clone was purified with a QuickExtract™ DNA Extraction Solution (Lucigen, UK), and then the genomic sequence of the targeted site was analyzed by Sanger sequencing. The deletion of the target protein expression was confirmed by western blotting.

#### Antibodies

The antibodies for LC3 (rabbit polyclonal, MBL, Japan), p62/SQSTM1 (rabbit polyclonal, MBL), NDP52 (rabbit polyclonal, GeneTex, CA), VPS33A (rabbit polyclonal, Novus, CO), Decorin (mouse monoclonal IgG, clone #115402, R&D, MN), β-Actin (mouse monoclonal IgG, clone #6D1, MBL), Myc tag (mouse monoclonal IgG, clone #My3, MBL), His tag (rabbit polyclonal, MBL), FLAG® tag (mouse monoclonal IgG, clone #M2, Merck), HA tag (mouse monoclonal IgG, clone #F-7, Santa Cruz) were used in western blotting, followed by anti-mouse or anti-rabbit IgG conjugated with HRP (Thermo Fisher). An HRP-conjugated anti-DYKDDDDK tag antibody (mouse monoclonal IgG, clone #1E6, Fujifilm-Wako, Japan) was also used for the detection of the FLAG® tag in some experiments. The anti-LC3 antibody was also used in In Cell Western, followed by Alexa 488-labelled rabbit IgG (Thermo Fisher) to detect endogenous LC3 on flow cytometry. The anti-Heparan Sulfate antibody (mouse monoclonal IgM, clone #10E4, AMSBIO, UK) was used for immunofluorescence analysis, which was followed by Alexa 647-labelled anti-mouse IgM antibody (Thermo Fisher).

#### Immunoblotting

Cells were directly lysed in sodium dodecyl sulfate (1× SDS) sample buffer (250 mM Tris-HCl (pH 6.8), 40% (w/v) glycerol, 6.2% (w/v) dithiothreitol, 8% (w/v SDS), bromophenol blue), heated at 95°C for 5 min, followed by shearing genomic DNA on a tube shaker at room temperature for 5 min. The treated samples were separated by SDS-PAGE and transferred onto polyvinylidene fluoride membranes (Merck). The membranes were blocked with 1% skimmed milk in TBS-T (Tris-buffered saline with 0.5% Tween 20) for 1 hr., followed by incubating with primary antibodies for 1 hr. at room temperature or 4°C overnight. After serial washings with TBS-T, the membrane was incubated with HRP-conjugated secondary antibody at room temperature for 1 hr. HRP signals on the membrane were visualized with Immobilon Forte (Merck) as a substrate. The chemiluminescence images were captured by ChemiDoc Touch (Bio-Rad, CA) and quantified with Image Lab Ver. 6.1.0 (Bio-Rad).

#### Autophagic flux assay

Autophagic flux assay was performed as described previously. Briefly, cells were seeded and cultured one day before the assay. The confluency of cells was adjusted approximately to 70% at seeding to achieve a confluent condition on the day of assay. The medium was replaced with pre-warmed fresh one with or without 125 nM Bafilomycin A1 (BafA1) (Cayman Chemicals, MI). After 2 hr. incubation, cells were washed with PBS twice and lysed in SDS sample buffer. The samples were resolved in 13% SDS-PAGE gel and then analyzed by immunoblotting using anti-LC3 and anti-ACTB antibodies.

#### Immunoprecipitation

Cells were transiently transfected using Effectene reagent (Qiagen) with the plasmids coding the tethering complex subunits. After 48 hr., cells were washed twice in cold PBS, then incubated in NP-40 Lysis buffer (150 mM NaCl, 1% NP-40, 50 mM Tris-HCl pH 8.0) with protease inhibitor cocktail (Merck) on ice for 15 min and collected by scraping. Cell lysates were centrifuged at 11,000 x g for 10 min at 4°C. Protein concentration was determined by Bradford assay (Bio-Rad). Equal amounts of proteins were used for the immunoprecipitation experiment. The cell lysates were precleared and immunoprecipitated with anti-Myc (MBL) antibodies at 4°C for 3 hr. Protein G Sepharose 4 Fast Flow beads (Cytiva, MA) were then added and incubated at 4°C with rotation overnight. The incubated beads were washed with NP-40 Lysis buffer four times and once with wash buffer (50 mM Tris-HCl, pH 8.0). The samples were boiled in 1× SDS sample buffer and were subjected to western blotting.

#### Immunofluorescence analysis

2 × 10^4^ of the skin fibroblasts were grown on collagen I-coated cover slips in 24-well plate overnight. The cells were treated or left untreated with 50 μM Triclabendazole for 24 hr. After incubation, the cells were fixed with 4% PFA for 15 min., then permeabilized/blocked with the cell staining buffer (0.05% Saponin / 10% FBS / 10 mM Glycine in PBS) at room temperature for 30 min. The cells were stained with anti-Heparan sulfate (10E4) at 4°C in moisture chamber overnight, followed by staining with Alexa 647-labelled anti mouse IgM secondary antibody and DAPI (Dojindo, Japan) at room temperature for 1hr. The cell staining buffer was used for all incubation and wash steps. The cover slips were mounted on glass slides and dried in a dark place overnight. The cell images were captured by LSM 900 confocal microscope (Zeiss, Germany) with a 40x objective lens.

#### Luciferase assay

The NanoLuc® (NLuc)-tagged VPS33A expressing HeLa cells were split in 96-well white plates. The cells were treated with the FDA-approved drugs for 24 hr. The supernatant was removed, and the cells were lysed with the Nano-Glo Assay Substrate (Promega). Their luminescence was determined by Varioskan (Thermo Fisher).

#### In cell western analysis

The tagged *SDC1*-expressing cells were seeded in 96-well black plates. After treating the cells with the FDA-approved drugs, the cells were washed with PBS and then fixed in 4% PFA (Nacalai-Tesque) for 5 min. The fixed cells were permeabilized in 0.1% Digitonin for 15 min at room temperature and stained with anti-HA antibody (GeneTex, CA) for 1 hr., followed by Alexa488-labelled anti-rabbit IgG secondary antibody with HCS-CellMask™ Deep Red (Thermo Fisher) for 60 min. After multiple washes with PBS, the fluorescence was measured by Infinite® PRO200 plate reader (TECAN, Switzerland).

#### Differential expression-function analysis using cytometry (DEFAC)

2.5 x 10^5^ VPS33A KO HeLa cells were seeded in 12-well plates. 2 μg/well of GFP-VPS33A expression plasmid or empty mock plasmid was transfected to the cell with PEI max. 125 nM of BafA1 was included for the last 2 hr. of incubation for the control group. 24 hr. after transfection, the analyte cells were trypsinized to have a single-cell suspension and then neutralized with complete DMEM. The cells were washed twice with PBS, followed by fixation with phosphate-buffered 4% PFA at room temperature for 5 min. After two washes, the fixed cells were permeabilized and blocked in a staining buffer (0.05% Saponin in 10% FBS/PBS) on ice for 30 min. The cells were stained with anti-LC3 antibody diluted with the staining buffer for 30 min, followed by staining with anti-rabbit IgG-Alexa488. The cells were washed and resuspended in the staining buffer with 7-AAD (Merck). The fluorescent signals were determined by FACSCanto™ II (BD Biosciences). 7-AAD-positive cells were excluded from the analyses as a dead cell population.

#### Electron microscopy

Cells were washed with PBS and then fixed in 2.5% glutaraldehyde in phosphate buffer for 120 min at room temperature. Subsequently, the cells were postfixed in 1% osmium tetroxide for 60 min at 4°C. After dehydration by passing through a series of graded concentrations of ethanol, the cells were embedded in Epon-resin for 3 days at 60°C. 70 nm ultrathin sections were made using EM UC7 ultramicrotome (Leica, Germany) and mounted on mesh grids. The sections were stained with 2% uranyl acetate for 20 min, followed by lead citrate staining for 5 min at room temperature. The samples were observed using a transmission electron microscope, JEM-1400 (JEOL, Japan), at an accelerating voltage of 80 kV. The aberrant endolysosomes were quantified using Fiji (ImageJ2).

#### ELISA

Enzyme-Linked Immunosorbent Assay (ELISA) for the determination of the cellular level of Heparan Sulfate was performed by using a pre-designed kit (CUSABIO, TX). Cells were harvested by scraping in PBS (-) after 2 washes. The cell suspension was disrupted (output 20%, alternate cycle of 1 sec. ON/1 sec. OFF, total 30 cycles, on ice) with a hand-held sonicator, SFX-150 (EMERSON, CT), and then centrifuged at 5,000 x g at 4°C for 5 min. The supernatants were removed as the ELISA samples. Protein concentration was determined by Bradford assay using BSA as a standard, and adjusted to 1 mg/mL with the sample diluent provided in the kit. The assay was performed according to the instructions of the company.

#### Mass spectrometry

The normal skin fibroblasts and the patient-derived skin fibroblasts were treated or left untreated with 10 μM of Triclabendazole for 3 days. The cells were collected by scraping and washed with cold PBS. The cells were disrupted by sonication as described above. The protein concentration was determined by Bradford assay and adjusted to 1.5 mg/mL with PBS. The same amount of the cell lysate was subjected to pre-processing for mass spectrometry. The sample was digested with Chondroitinase B (For DS detection), Heparinase I/III (For HS detection), and Keratanase II (For KS detection) separately at 37°C for 18 hr. Methanol was added to the samples for deproteinization and centrifuged, and the supernatants were collected and used as the LC-MS sample. The sample was analyzed by Nexera™-XR/LCMS-8060NX (SHIMADZU, Japan).

#### Animal experiments

*Vps33a* KI mice were generated by genome editing with the CRISPR/Cas9 system. Guide RNA (crRNA) against the genomic sequence 5' AGCAT AACCG CTGTA CACAT 3' (anti-sense chain of *Vps33a* gene) of the mouse *Vps33a* gene was synthesized and mixed with equimolar amounts of TracrRNA and annealed by heating at 95°C for 5 min. followed by gradual cooling. The crRNA and TracrRNA were mixed with Cas9 Nuclease (IDT, IA) and donor oligo for introducing p.R500W mutation: 5’ CAGAA CCCCA CGGAC ATATC GTATG TGTAC AGTGG GTATG CCCCA CTCAG TGTGT GGCTT GCCCA GCTGC TTTCC CGGCC TGGCT GGCGG AGCAT CGAGG 3’, and allowed to stand at room temperature for 20 minutes to form CRISPR/Cas9 complex. The mixture of prepared CRISPR/Cas9 complex and the donor oligo was introduced into fertilized eggs derived from C57 BL6/J mice (SLC, Japan) by NEPA21 electroporator (NEPAGENE, Japan). The next day, fertilized eggs that had progressed normally to the 2-cell stage were implanted into the oviducts of pseudopregnant ICR female mice. Since the founder male mouse with the KI allele was obtained as a mosaic, sperm was further collected from this male mouse and used for the second IVF. To reduce the effect of off-target mutations, the heterozygote mice obtained by the second IVF were backcrossed to WT C57 BL/6J mice for four generations before being used in the current experiment. The mice were maintained in a conventional environment at the animal facility of our school.

#### RT-PCR

Total RNA was extracted from intact or Triclabendazole-treated HeLa cells using FastGene™ RNA Basic kit (NIPPON Genetics, Japan) according to the manufacturer’s instructions. 1 μg of total RNA from each sample was subjected to reverse transcription using PrimeScript™ 1^st^ strand cDNA Synthesis Kit (TaKaRa, Japan). Quantities of the target transcripts were determined by the amplification curves using SYBR™ Green qPCR Master Mix (Thermo Fisher) obtained by QuantStudio® 5 real-time PCR system (Thermo Fisher). The target sequence-specific primer sets were used for the amplifications; *SDC1*: 5’ TGGGG ATGAC TCTGA CAACT TC 3’ and 5’ TGCGT GTCCT TCCAA GTGG 3’, *DCN*: 5’ AAGAT GAGGC TTCTG GGATA GG 3’ and 5’ TCGAA GATGG CATTG ACAGC 3’, *TFEB*: 5’ GAGCG GCAGA AGAAA GACAA TC 3’ and 5’ GATCA GCATT CCCAA CTCCT TG 3’, *TFE3*: 5’ TGATC CTGAC AGCTT CTACG AG 3’ and 5’ ACGAT GCAGA GAGTG TAGCT G 3’, *MITF*: 5’ GGCTA TGCTT ACGCT TAACT CC 3’ and 5’ ACGCT CGTGA ATGTG TGTTC 3’, *TFEC*: 5’ TGGCA CGGTT GATTT AGGTG 3’ and 5’ AGACA CAGTC AGTTG TTGGC 3’, *GAPDH*: 5’ CAATG ACCCC TTCAT TGACC 3’ and 5’ GACAA GCTTC CCGTT CTCAG 3’.

### Quantification and statistics analysis

Parametric comparisons between two groups with equal variances were conducted using Student’s *t*-test, whereas Welch’s *t*-test was applied for comparisons involving unequal variances. For multiple group comparisons of normally distributed data, one-way ANOVA was employed, followed by Tukey’s *post hoc* test for pairwise analyses. In cases where the data deviated from normality, the Kruskal–Wallis test was used, with subsequent pairwise comparisons performed using the Mann–Whitney U test adjusted by the Holm method. The chi-square test was applied to test the bias in the distribution of mouse fetus genotypes. All statistical analyses were performed using EZR (Easy R), a graphical user interface for R Commander, which is tailored for statistical functions commonly used in biostatistics (The R Foundation for Statistical Computing, Austria).^58^ A *p*-value of less than 0.05 was considered statistically significant for all tests (∗ *p* < 0.05, ∗∗ *p* < 0.01, ∗∗∗ *p* < 0.001). Statistical details are provided in the figure legends, and reported values are presented as means and standard deviations (SD).

## Supplemental information

Document S1. Figures S1–S7, Table S1, and Uncropped Membrane Images

Data S1. An Excel file containing the raw data of FDA-approved drug screening
