# Supplementary material for: Triclabendazole suppresses cellular levels of glycosaminoglycan—A potential therapeutic agent for mucopolysaccharidoses and related diseases
Source: iScience. 2025 Jul 18;28(8):113118. doi: 10.1016/j.isci.2025.113118 (PMC12355420; doi:10.1016/j.isci.2025.113118)

iScience, Volume *28*

Supplemental information

Triclabendazole suppresses cellular levels of glycosaminoglycan—A potential therapeutic agent for mucopolysaccharidoses and related diseases

Seigo Terawaki, Filipp Vasilev, Viktoriia Sofronova, Misa Tanaka, Yoshiko Mori, Rina Iwata, Takahito Moriwaki, Toshiharu Fujita, Nadezhda Maksimova, and Takanobu Otomo

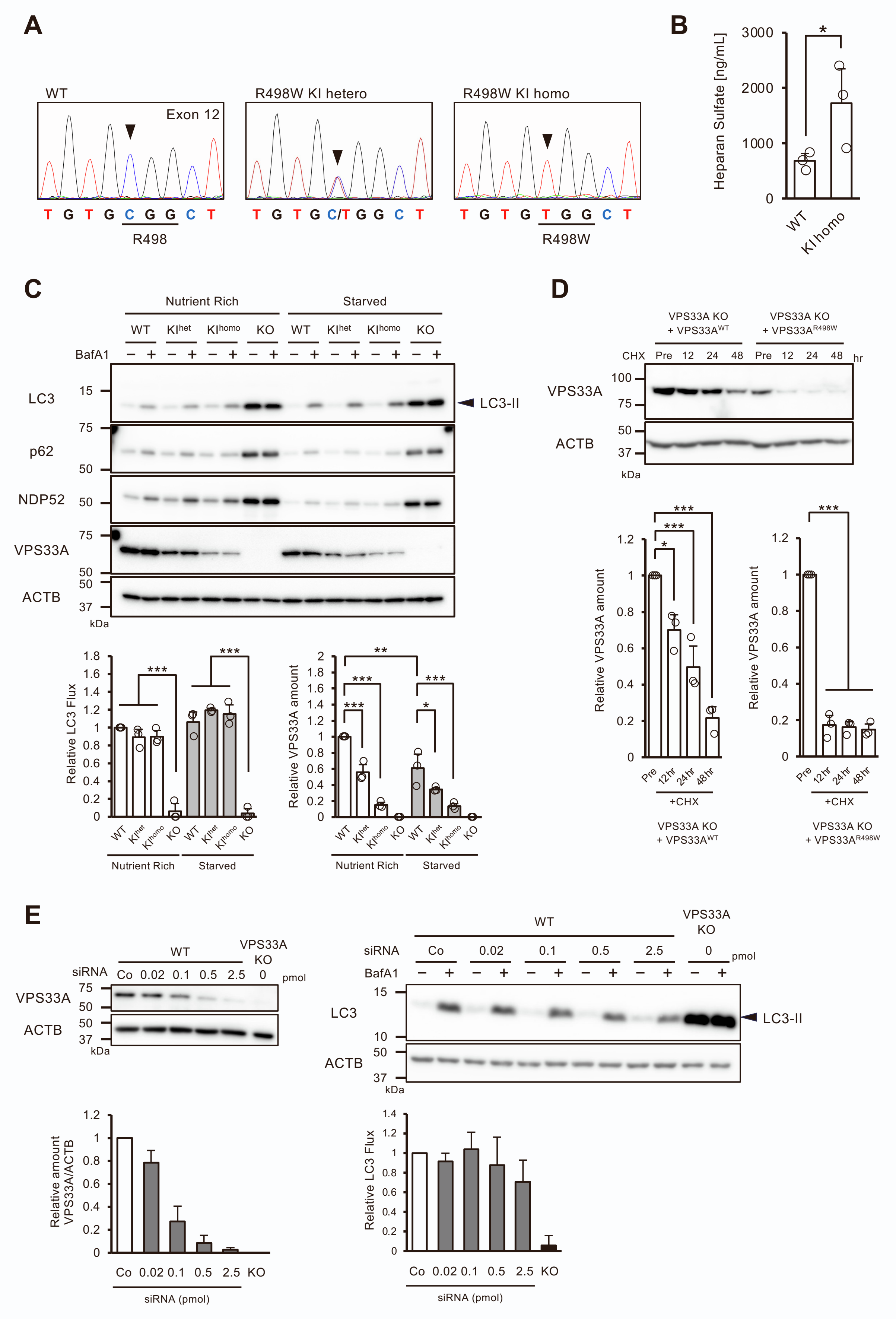
A
WT
Exon 12
R498W KI hetero
R498W KI homo
T G T G C G G C T
R498
T G T G C/T G G C T
T G T G T G G C T
R498W
B
Heparan Sulfate [ng/mL]
WT
KI homo
C
Nutrient Rich
Starved
WT KIhet KIhomo KO WT KIhet KIhomo KO
BafA1
LC3
LC3-II
p62
NDP52
VPS33A
ACTB
kDa
Relative LC3 Flux
Relative VPS33A amount
D
VPS33A KO + VPS33AWT
VPS33A KO + VPS33AR498W
CHX Pre 12 24 48 hr
VPS33A
ACTB
kDa
Relative VPS33A amount
Pre 12hr 24hr 48hr
+CHX
E
siRNA Co 0.02 0.1 0.5 2.5 0 pmol
WT
VPS33A KO
VPS33A
ACTB
kDa
BafA1
LC3
LC3-II
ACTB
Relative amount VPS33A/ACTB
Relative LC3 Flux
siRNA (pmol)

Figure S1. R498W mutation of VPS33A shortens its half-life without affecting autophagic flux.

(**A**) The electropherograms from Sanger sequencing at the targeting site for the p.R498W mutation in *VPS33A* KI heterozygote and homozygote cells. The positions where bases were replaced by gene editing (or the original position in WT) are indicated by arrowheads.

(B) Accumulation of Heparan Sulfate in the *VPS33A* KI homozygote cells. The amount of Heparan Sulfate in the cell lysate of HeLa WT and the KI homozygote was determined by competitive ELISA. Data are shown as mean and S.D. from 3 independent preparations. The small circles represent the data for each. Statistical analysis was done by *Student's t-test*.

(C) Autophagy activity in the *VPS33A* KI heterozygote and homozygote cells. HeLa WT, $VPS33A^{p.R498W}$ KI heterozygote, KI homozygote, and *VPS33A* KO cells were cultured with or without 125 nM BafA1 under nutrient-rich or starved conditions. The cell lysates were analyzed for LC3, p62, NDP52, VPS33A, and β-Actin (ACTB). Representative blot images are shown. Quantitative analyses for LC3 and VPS33A from 3 independent experiments are shown in the lower panels. Each LC3-II band intensity was normalized to its ACTB amount, then the LC3 flux was calculated as follows: LC3-II $^{BafA1+}$ – LC3II $^{non\text{-}treat}$ / LC3II $^{BafA1+}$. LC3 flux and protein levels of VPS33A are shown as relative amounts with the WT untreated sample as 1. The small circles represent the data from each experiment, and the bars show the mean and S.D. of the three independent experiments.

(**D**) CHX pulse-chase assay of WT and the MPSPS mutant VPS33A protein. The *GFP*-tagged *WT* or the MPSPS mutant *VPS33A* gene was introduced into *VPS33A* KO cells and then expressed for 24 hr. The transfected cells were further cultured in the presence of 50 μg/mL CHX, and the VPS33A protein level was chased up to 48 hr. Quantitative analyses of the VPS33A amount for WT (left half) and the mutant VPS33A (right half) from 3 independent experiments are shown in the lower panels. Statistical analysis was performed by *One-way ANOVA*, followed by the *Tukey test*.

(**E**) Effect of VPS33A gene knockdown on autophagy flux. siRNA against VPS33A was introduced into wild-type HeLa cells at different doses as indicated. After 48 hr., the cells were cultured in the presence and absence of 125 nM BafA1 for 2 hours, and cell lysates were collected and analyzed for VPS33A protein level (left panel) and LC3 flux (right panel) by western blotting. Data are shown as mean and S.D. of three independent experiments in the

bottom panels. The sample from *VPS33A* KO cells was loaded on the rightmost lane(s) as a negative control.

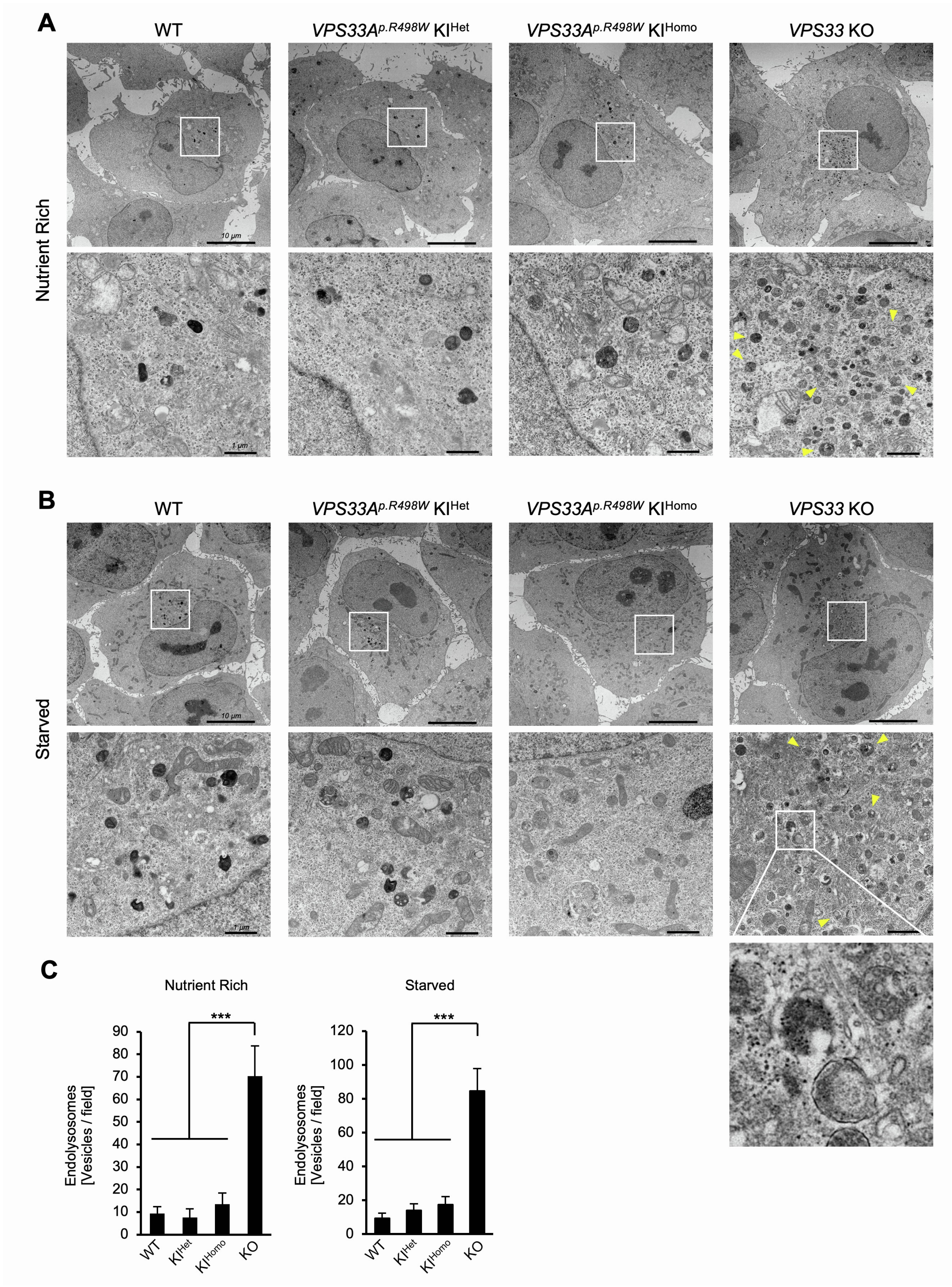

Figure S2. Electron microscopic analysis of *VPS33A* KO and *VPS33A$^{p.R498W}$* KI cells.

(A and B) Transmission electron micrographs of HeLa WT, *VPS33A$^{p.R498W}$* KI heterozygote, KI homozygote, and *VPS33A* KO cells under nutrient-rich (A) or starved conditions (B).

Representative images are shown. Enlarged images in the white square are shown in the lower panel of each. Aberrant endolysosomes in *VPS33A* KO cells are indicated with yellow arrowheads. Bars in lower magnification: 10 μm, Bars in higher magnification: 1μm.

(C) Quantitative analyses of the endolysosome for (A) and (B). The number of electron-dense endolysosomes was counted from 5 fields for each cell using Fiji, and the bar graphs represent the mean values and S.D. of them. Left panel: under nutritional conditions (A), right panel: under starvation conditions (B).

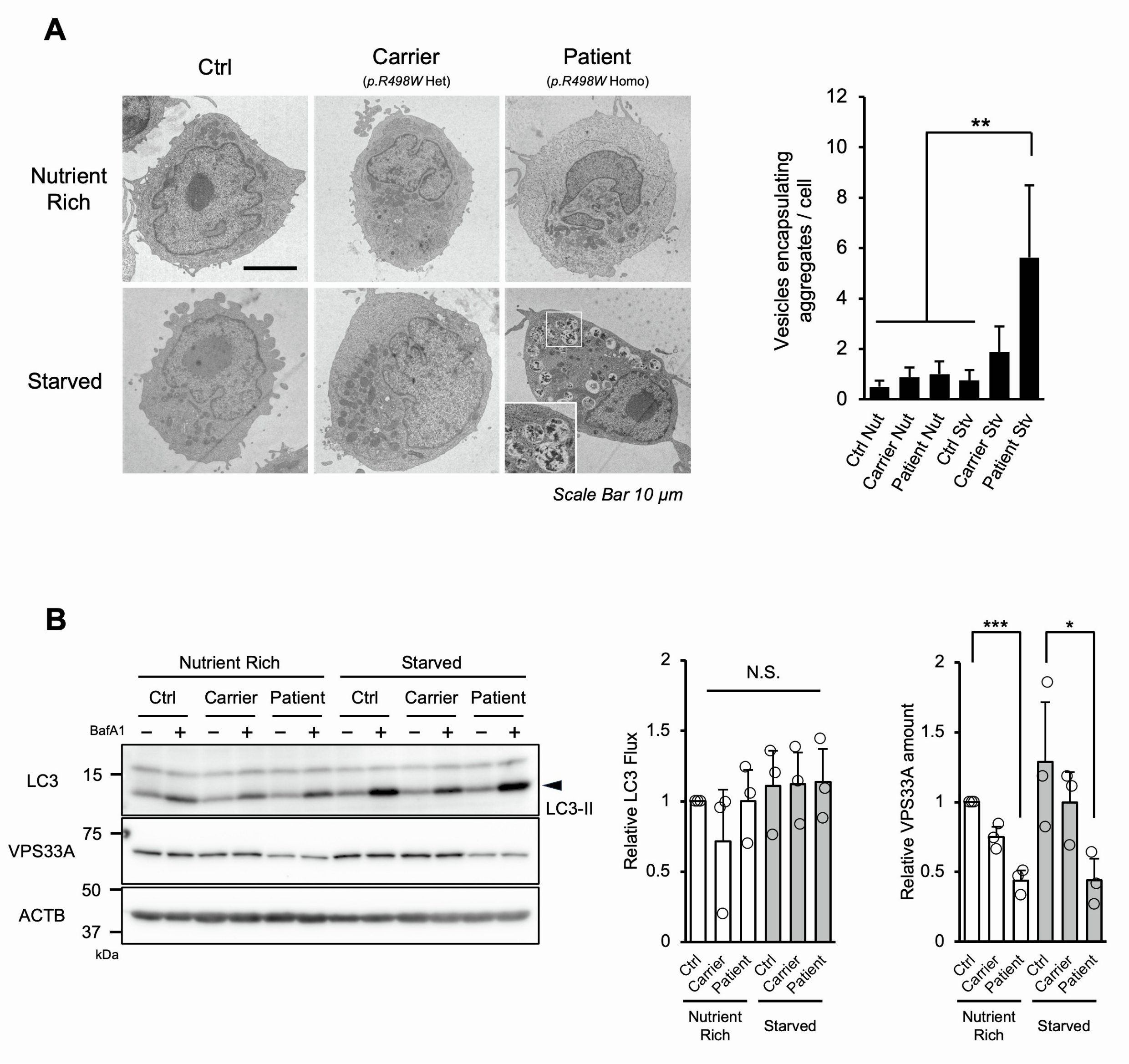

Figure S3. Electron microscopic analysis of lymphoblastoid cell lines (LCL) derived from MPSPS patients.

(A) Transmission electron micrographs of healthy control, MPSPS carrier (heterozygote), and MPSPS patient-derived LCLs under nutrient-rich or starved conditions. Representative images are shown. An enlarged image in the white square is shown in the inset. High-density endolysosomes were manually counted from 8 cells for each condition. The bar graph represents the average and S.D. of the cells counted.

(B) Autophagy flux of the LCLs. The cells were cultured in the absence or presence of 125 nM BafA1 for 2hr. under nutrient-rich or starved conditions. The protein amounts for LC3, VPS33A, and ACTB were evaluated by western blotting. Representative blots are shown. The Quantitative analyses for LC3 flux and VPS33A amount are shown on the right side. The bar

graphs represent the average and S.D. from 3 independent experiments. The circles indicate each value for the repeated experiments. Ctrl: Control LCL from a healthy individual.

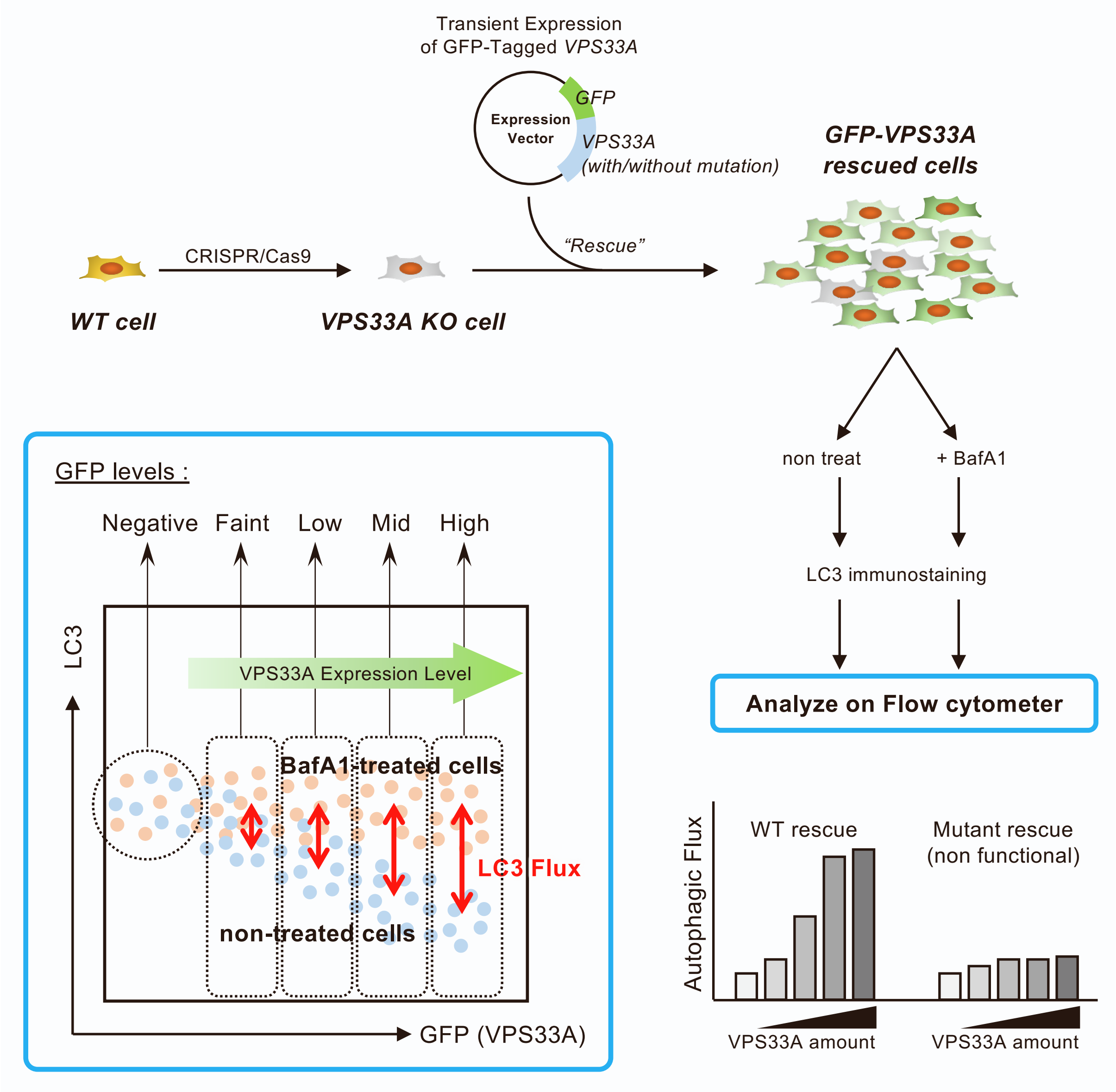

Figure S4. Principle of Differential expression-function analysis using cytometry (DEFAC).

Schematic workflow of the DEFAC. The *VPS33A* KO cell was established by the CRISPR/Cas9 method. *GFP*-tagged *VPS33A* is transiently re-expressed in the KO cells. The analyte cells need to be divided into BafA1-treated and untreated groups if the DEFAC is applied to the LC3 Flux Assay. The endogenous LC3 is fluorescently labeled after fixation and permeabilization and then analyzed on a flow cytometer (Flow chart). Cells were classified into several populations according to the GFP level as desired. The LC3 flux of each population can be calculated by subtracting the MFI of non-treated samples from the BafA1-treated sample of the same GFP level population. For the case of VPS33A, LC3 Flux increases along with the functional VPS33A protein level recovery. (Lower left panel marked with blue

rectangle.) If the non-functional VPS33A is introduced to the VPS33A KO cells, the LC3 flux will not recover even if the protein level increases (right bottom panel).

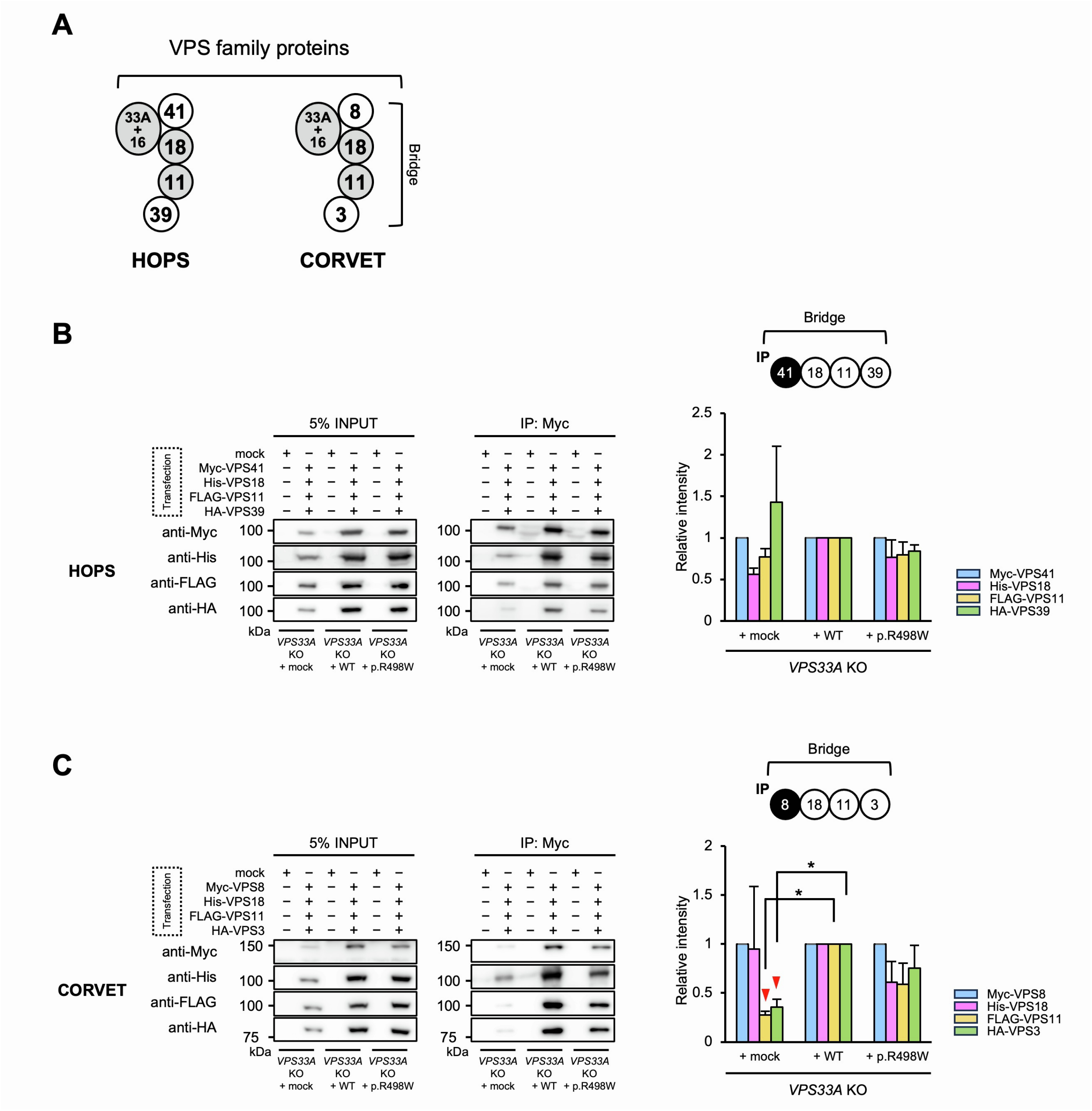

Figure S5. The effects of the VPS33A MPSPS mutation on the bridge formation of HOPS and CORVET.

(A) Schematic representation of the tethering complexes (HOPS and CORVET).

(B and C) Immunoprecipitation analyses for HOPS (B) and CORVET (C) bridge formations in the *VPS33A KO* cells rescued by WT VPS33A or the mutant VPS33A$^{p.R498W}$. *VPS33A* KO cells were transfected with differently tagged bridge subunit expression vectors in combination with *WT* or the mutant *VPS33A*. HOPS and CORVET bridge formation was evaluated by the detection of each tag attached to the corresponding subunits in the immunoprecipitates with anti-Myc Tag antibody. The relative amount of each subunit was normalized to the signal

intensity from the samples rescued by WT VPS33A as 1. The failed bridge formation observed in *VPS33A* KO cells is indicated with red arrowheads. The bar graphs representing the average and S.D. from 3 independent experiments are shown on the right side.

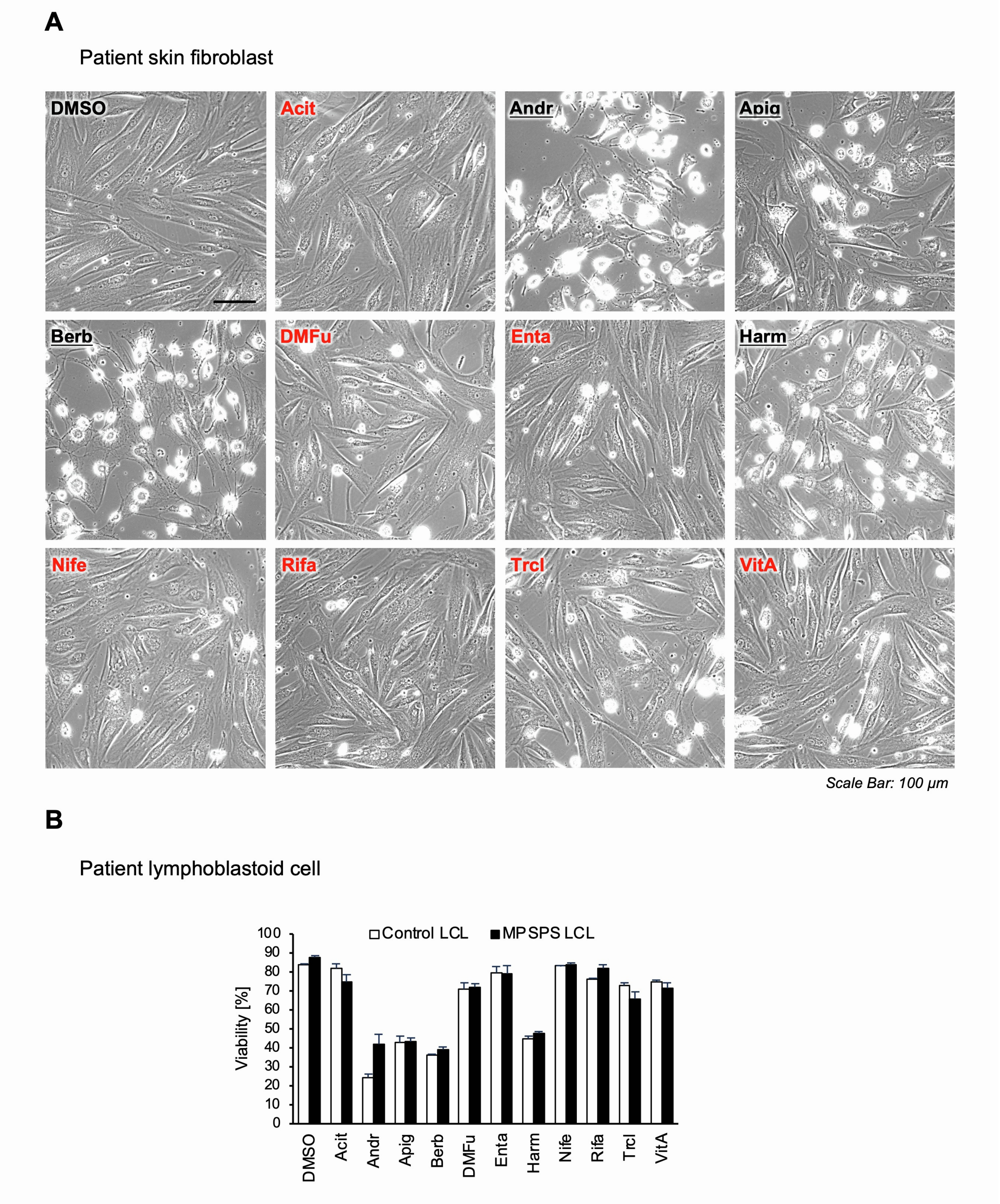

Figure S6. Toxicity checks of the selected drugs using MPSPS patient-derived cells.

(A) Representative images of the phase contrast microscope of the MPSPS patient skin fibroblast treated with the indicated drugs. The patient-derived skin fibroblasts were treated with the indicated drug at 50 μM for 24 hr. The drugs that represent high toxicity are underlined. The other drugs shown with red characters were selected for further screening, based also on the results of (B).

(B) The toxicity of the eleven drugs, same as in (A), on patient-derived or control lymphoblastoid cell lines (LCL). The viability of the cells was evaluated by trypan blue staining after 24 hr. drug treatments. The relative viability was calculated from 3 independent wells of each condition and is shown as a bar graph with the average and S.D.

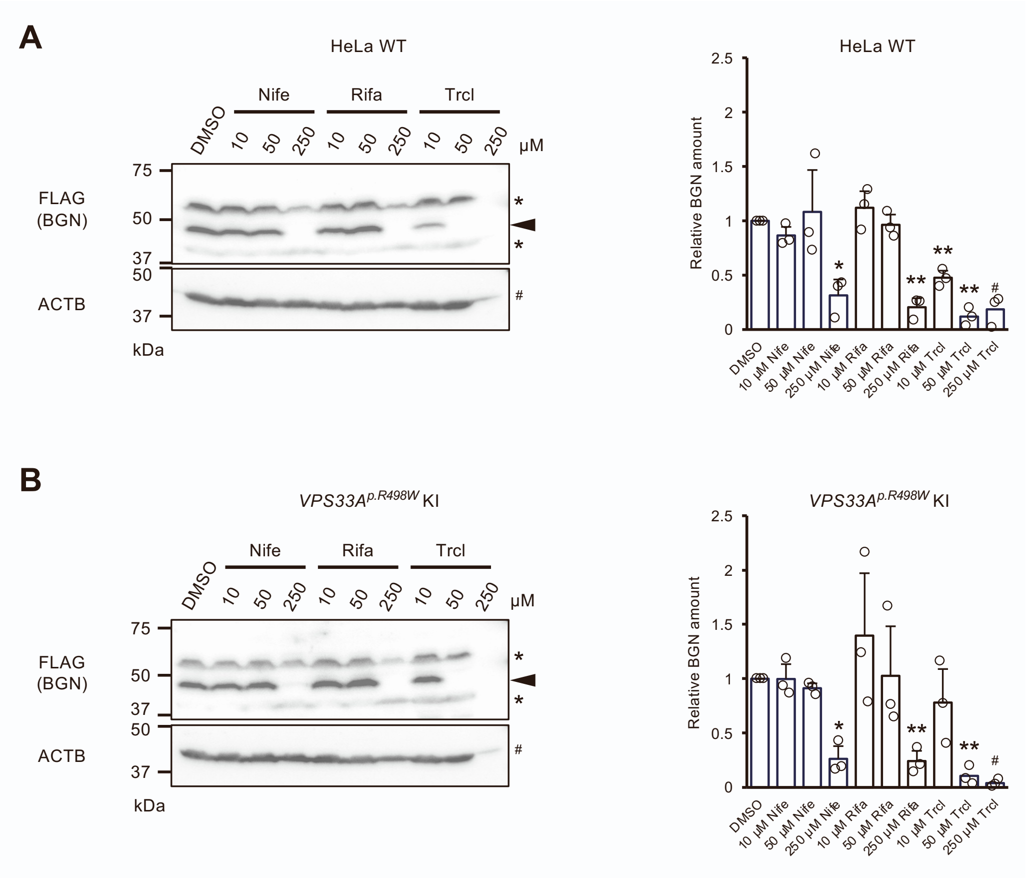

Figure S7. Dose effect of the selected drugs on the GAG core protein level.

(A and B) Western blot analyses for BGN levels in HeLa WT (A) or *VPS33A$^{p.R498W}$* KI cells (B). The cells were transfected with *FLAG-tagged BGN* and expressed for 24 hr. The cells were treated with the indicated concentration of Nifedipine (Nife), Rifaximin (Rifa), and Triclabendazole (Trcl) for an additional 24 hr. The cell extracts were analyzed for FLAG tag (BGN) and β-Actin (ACTB) amount by western blotting. Representative blots are shown. The arrowhead indicates BGN bands, and the asterisk represents non-specific bands. The cells treated with 250 µM Triclabendazole were detached from the dishes, resulting in poor sample recovery as reflected by the actin blot (#). The amount of BGN was quantified by densitometry and normalized to that of ACTB intensity. The bar graph on the right side represents the average and S.D. from 3 independent experiments.

**Chemical Name**	**MW**	**Abbreviation** in this manuscript	**Target Molecule or Pathway**	**Application**
Acitretin	326.429	Acit	RAR/RXR	Resistant psoriasis
Andrographolide	350.45	Andr	IκB/IKK	
Apigenin	270.0528	Apig	PI3K/Akt/mTOR	Anti-anxiety agent
Azaguanine-8	152.11		tRNA, Translation	
Berberine Sulfate	384.4*	Berb	ROS, DNA Topoisomerase	Anti-diarrheal
Ciclopirox	207.269		ATPase	Anti-fungal
Ciclopirox ethanolamine	268.35		ATPase	Anti-fungal
Dimethyl Fumarate	144.127	DMFu	KEAP1-Nrf2	Oral treatments for psoriasis, Multiple sclerosis, Immunosuppressants
Entacapone	305.286	Enta	COMT, Histone methyltransferase	Parkinson's disease
Etoposide	588.56		Topoisomerase II	
Harmine hydrochloride	248.71	Harm	Monoamine Oxidase	
Nifedipine	346.335	Nife	Dihydropyridine calcium channel	Hypertension and angina pectoris treatment
Oxaliplatin	397.285		DNA	
Rifaximin (Xifaxan)	785.879	Rifa	Akt/mTOR, p38MAPK/NF-κB	Rifamycin-type antibacterial drug
Rucaparib (AG-014699, PF-01367338)	323.371		PARP/H6PD	
Teniposide	656.65		Topoisomerase II	
Triclabendazole	359.658	Trcl	Excretory secretory (ES)	Anthelmintic
Vitamin A	286.4516	VitA	RAR/RXR	Nutritional supplement

MW: Molecular Weight

Gray columns indicate anticancer drugs and topical medicines.

* $C_{20}H_{18}NO_4 \cdot 1/2\ SO_4$

Table S1. FDA-approved Drugs for decreasing GAG and maintaining VPS33A stability

Supplemental Figure
Uncropped Membrane Images

Membrane margin
Cropped area

Figure 3

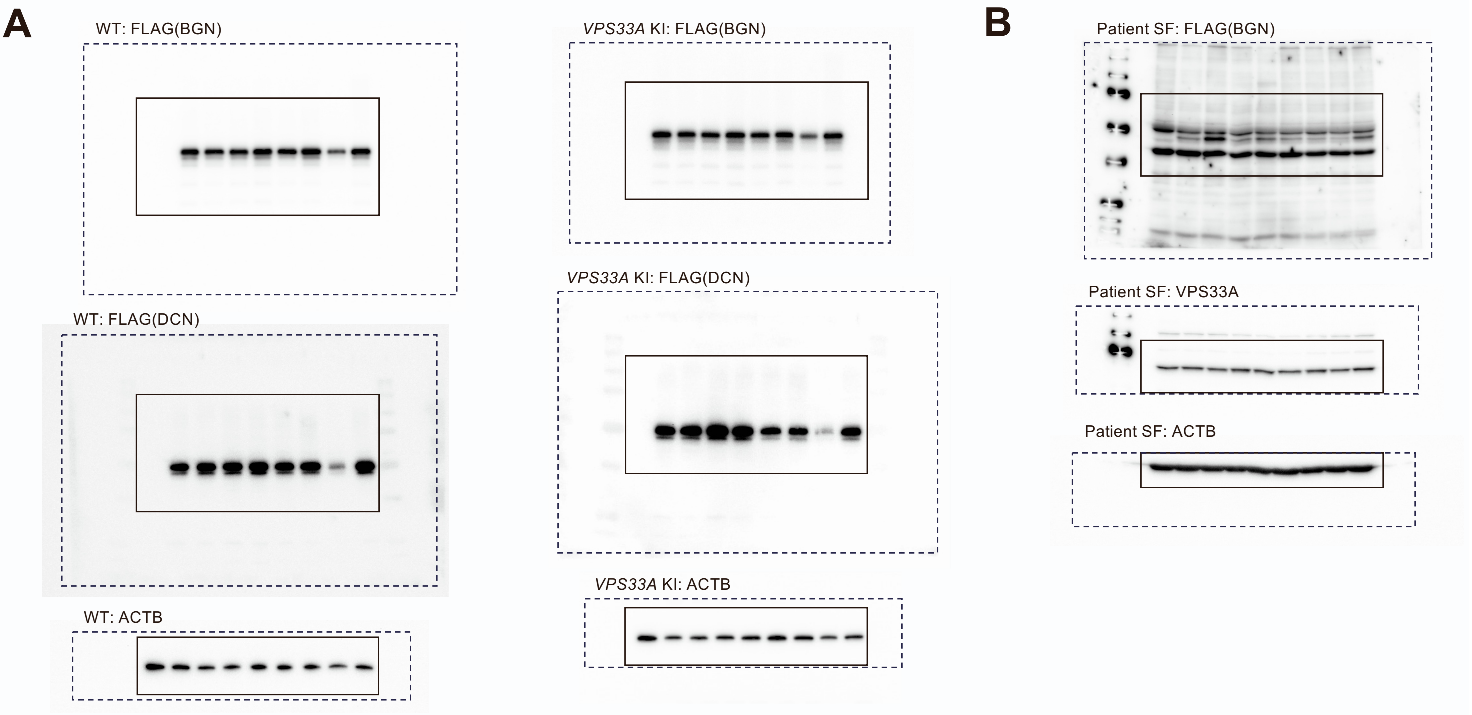

Figure 4

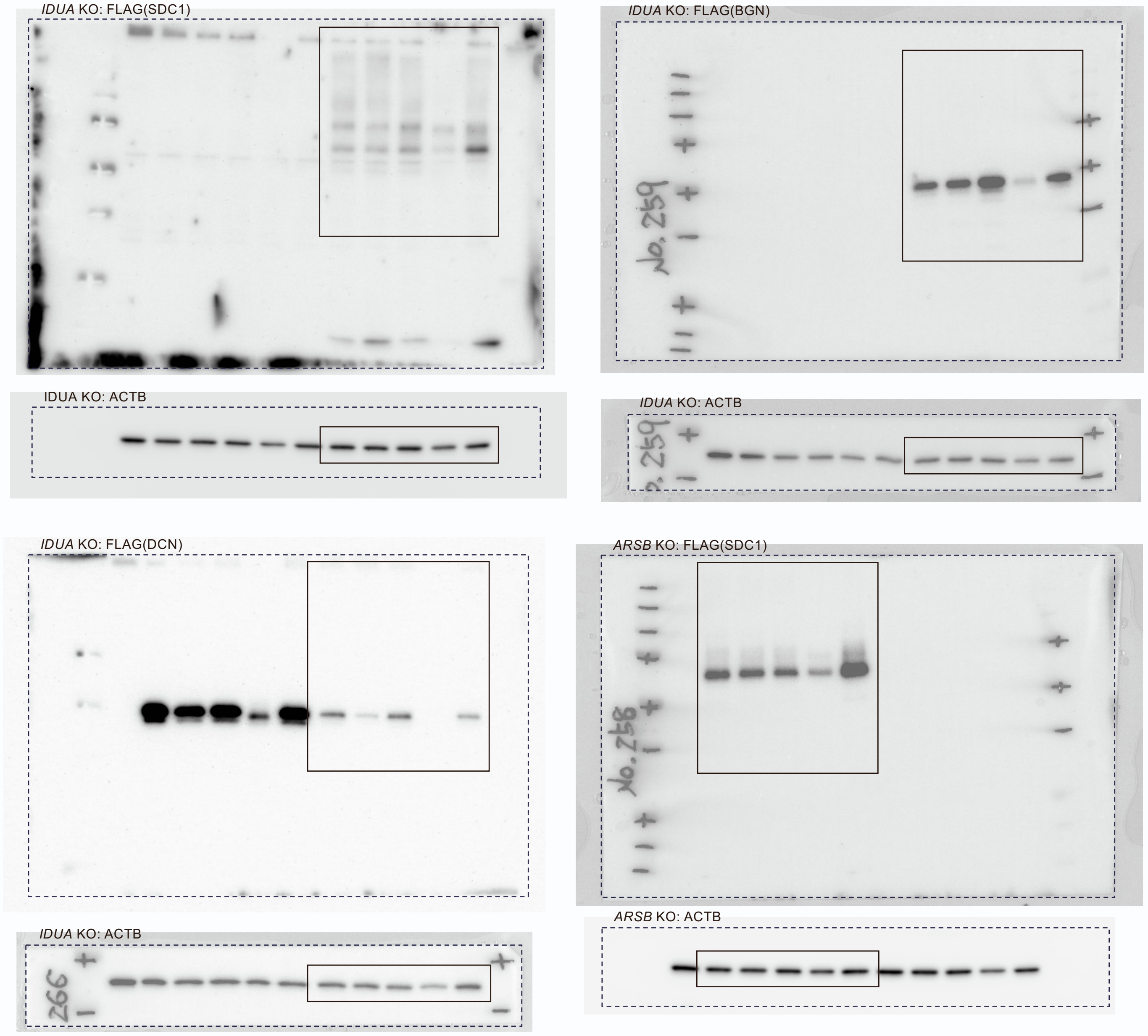

Figure 4

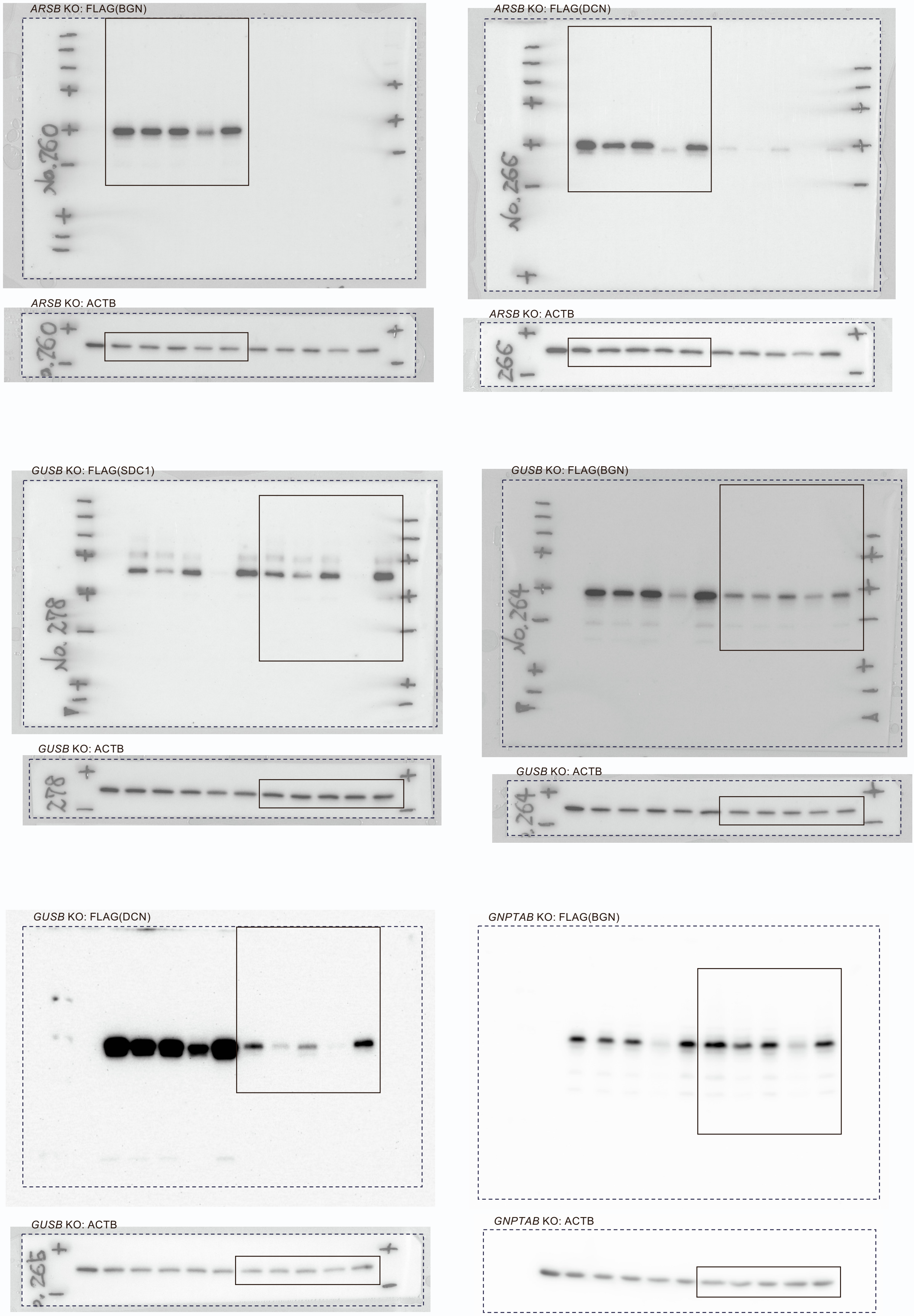
Membrane margin
Cropped area
ARSB KO: FLAG(BGN)
ARSB KO: ACTB
ARSB KO: FLAG(DCN)
ARSB KO: ACTB
GUSB KO: FLAG(SDC1)
GUSB KO: ACTB
GUSB KO: FLAG(BGN)
GUSB KO: ACTB
GUSB KO: FLAG(DCN)
GUSB KO: ACTB
GNPTAB KO: FLAG(BGN)
GNPTAB KO: ACTB

Membrane margin

Cropped area

Figure 6

A

WT: DCN

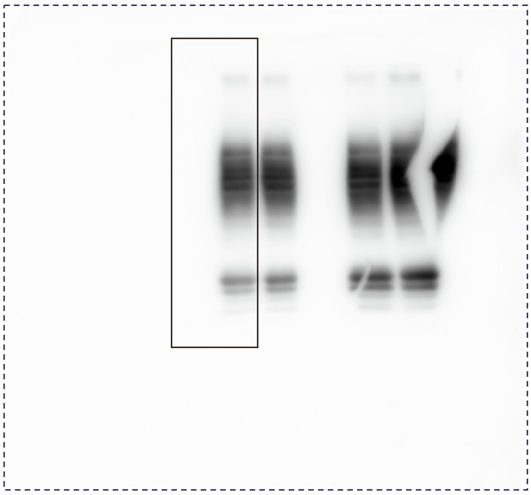

B

Skin Fibroblast: DCN

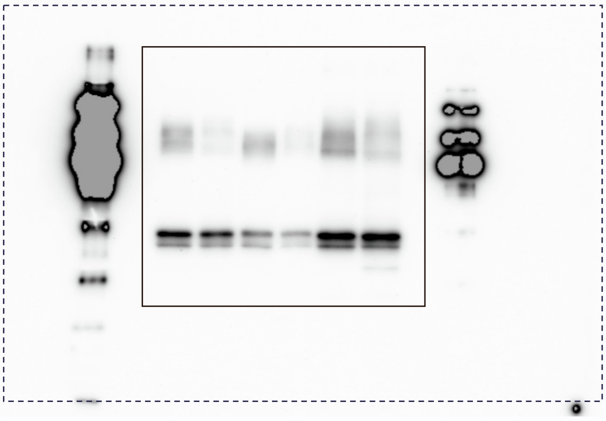

Skin Fibroblast: ACTB

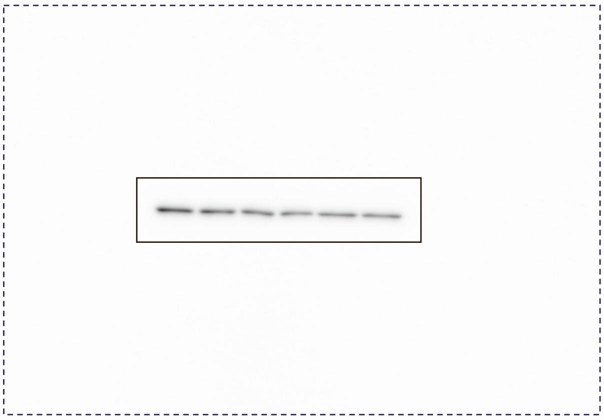

Figure 7

B

WT: FLAG(SDC1)

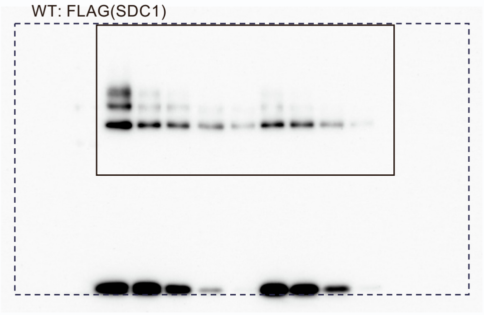

WT: ACTB

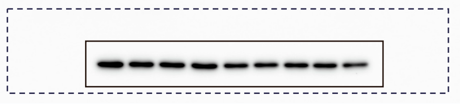

WT: FLAG(DCN)

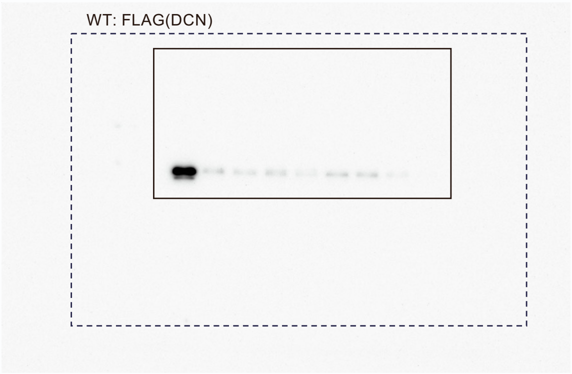

WT: ACTB

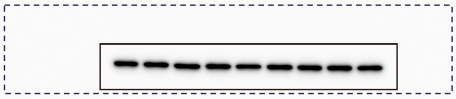

Supplement: Document S1. Figures S1–S7, Table S1, and Uncropped Membrane Images [file mmc1.pdf]
